# *De novo* Sequencing of the Leaf Transcriptome Reveals Complex Light-Responsive Regulatory Networks in *Camellia sinensis* cv. *Baijiguan*

**DOI:** 10.3389/fpls.2016.00332

**Published:** 2016-03-21

**Authors:** Quanjin Wu, Zhidan Chen, Weijiang Sun, Tingting Deng, Mingjie Chen

**Affiliations:** ^1^Department of Tea Science, College of Horticulture, Fujian Agriculture and Forestry UniversityFuzhou, China; ^2^Department of Tea Science, Anxi College of Tea Science, Fujian Agriculture and Forestry UniversityFuzhou, China; ^3^Haixia Institute of Science and Technology, Fujian Agriculture and Forestry UniversityFuzhou, China

**Keywords:** light-shading, *Camellia sinensis*, differentially expressed genes, yellow phenotype, chloroplast development, chlorophyll synthesis, antioxidant enzymes

## Abstract

Tea plants (*Camellia sinensis* L.) possess high genetic diversity that is important for breeding. One cultivar, *Baijiguan*, exhibits a yellow leaf phenotype, reduced chlorophyll (Chl) content, and aberrant chloroplast structures under high light intensity. In contrast, under low light intensity, the flush shoot from *Baijiguan* becomes green, the Chl content increases significantly, and the chloroplasts exhibit normal structures. To understand the underlying molecular mechanisms for these observations, we performed *de novo* transcriptome sequencing and digital gene expression (DGE) profiling using Illumina sequencing technology. *De novo* transcriptome assembly identified 88,788 unigenes, including 1652 transcription factors from 25 families. In total, 1993 and 2576 differentially expressed genes (DEGs) were identified in *Baijiguan* plants treated with 3 and 6 days of shade, respectively. Gene Ontology (GO) and pathway enrichment analyses indicated that the DEGs are predominantly involved in the ROS scavenging system, chloroplast development, photosynthetic pigment synthesis, secondary metabolism, and circadian systems. The light-responsive gene *POR* (protochlorophyllide oxidoreductase) and transcription factor *HY5* were identified. Quantitative real-time PCR (qRT-PCR) analysis of 20 selected DEGs confirmed the RNA-sequencing (RNA-Seq) results. Overall, these findings suggest that high light intensity inhibits the expression of photosystem II 10-kDa protein (*PsbR*) in *Baijiguan*, thus affecting PSII stability, chloroplast development and chlorophyll biosynthesis.

## Introduction

Tea plants (*Camellia sinensis* L.), whose leaves are used to make a traditional beverage consumed worldwide, possess great genetic diversity, with some cultivars exhibiting unique leaf colors. Leaf albinism, a common phenomenon in many ornamental and crop species, is caused by either genetic mutations or dramatic environmental factors, such as low temperature or high light intensity (Peng et al., 2012; Song et al., 2014). Several albino tea plant mutants have been identified in China; these mutants are classified into two categories: temperature-sensitive and light-sensitive mutants (Du et al., 2008). *Anji Baicha* is a temperature-sensitive tea cultivar; when the environmental temperature is below 20°C, the newly developed young shoots of this cultivar exhibits a white phenotype because of defective chloroplasts and reduced chlorophyll (Chl) content (Du et al., 2008; Ma et al., 2012). In contrast, *Baijiguan* is a light-sensitive tea cultivar; under high light intensity, the newly developed leaves of this cultivar exhibit a yellow phenotype, whereas if this cultivar is transferred to low intensity light, its yellow shoots turn green within several days. Temperature-sensitive albino tea plants have been extensively studied (Du et al., 2009; Li et al., 2011; Ma et al., 2012; Wei et al., 2012; Xiong et al., 2013; Feng et al., 2014); however, few studies regarding light-sensitive albino tea varieties have been conducted.

Previous studies have demonstrated that the albino or yellow phenotype can result from multiple factors, including Chl biosynthesis or degradation (Lin et al., 2014), tetrapyrrole synthesis for heme and phytochromobilin (Yaronskaya et al., 2003), carotenoid biosynthesis, or degradation (Qin et al., 2007; Wang et al., 2009), chloroplast development and biogenesis (Peng et al., 2012; Su et al., 2012), signal transduction during chloroplast development (Sheng et al., 2014), disease resistance (Nasir and Riazuddin, 2008), reduced light-harvesting Chl proteins (LHCPs) (Asakura et al., 2008), and photo-oxidative stress (Miura et al., 2010; Zhan et al., 2014). Digital gene expression (DGE) analysis of the tea cultivar *Anji Baicha* identified several genes involved in Chl biosynthesis or chloroplast development (Ma et al., 2012). Gene expression analysis identified that several photosynthesis-related genes were found to be suppressed during the albino stage, but increased dramatically when the shoots turned green. These differentially expressed genes (DEGs) include violaxanthin de-epoxidase (*VDE*), light harvesting Chl a/b-binding protein (*Lhcb*), Rubisco small subunit (*RbcS*), and Mg-chelatase subunit H (*ChlH*) (Ma et al., 2012). The albino phenotype of *Anji Baicha* has been reported to be caused by dysfunctional Chl biosynthesis (Ma et al., 2015). These studies have revealed some of the molecular mechanisms of leaf color formation in temperature-sensitive cultivars. In contrast, few transcriptome analyses of light-sensitive tea cultivars have been performed. The light-sensitive yellow leaf phenotype is a valuable trait that can be used to explore the molecular mechanisms underlying pigment metabolism, chloroplast development and leaf color formation. Therefore, we conducted RNA sequencing (RNA-Seq) and DGE analysis of a light-sensitive cultivar. The DEGs were assigned to diverse functional categories, and Gene Ontology (GO) annotation and pathway analyses revealed many light- and chloroplast-enriched categories. Twenty putative genes related to the light-induced yellow phenotype were selected for quantitative real-time PCR (qRT-PCR) analysis, the qRT-PCR results were in consistent with the DGE data. This study reveals the dynamic changes in gene expression in response to shade treatment and provides valuable insights into the genetic and genomic regulation of chloroplast development and chlorophyll biosynthesis in *C. sinensis*.

## Materials and methods

### Plant materials and sample preparation

*C. sinensis* (L.) O. Kuntze cv. *Baijiguan* was planted in the Germplasm Tea Repository (Imperial Tea Garden) at the Tea Research Institute, Wuyi Mountain, China. Compared with other green tea cultivars, *Baijiguan* exhibits a yellow leaf phenotype and significantly reduced Chl content. Fully expanded second leaves of this cultivar were collected from the flush shoots at the one-bud and four-leaf stages. For the light-shading experiment, half of the second leaf was covered with aluminum foil for 3–6 days, from October 10-16, 2013. After the shade treatment, the two parts of the leaf were separately harvested. All samples were divided into three portions. One portion was immediately frozen in liquid nitrogen, and then stored at −80°C for RNA isolation. The second portion was cut into thin strips and immediately fixed in glutaraldehyde solution (2.5%) for ultra-structural characterization. The third portion was used for pigment and enzyme activity assays. Pooled leaf samples (three leaves per plant) were collected from three randomly selected tea plants.

For light and temperature treatments, 2-year-old tea plants were grown in potting soil in MGC-350HPY-2 climatic-controlled cabinets (Shanghai Yiheng Instruments Co., Ltd., Shanghai, China). The temperature was set to 25°C during the light period (12 h) and to 15°C during the dark period, with 75% humidity (Figures S1A–D). For the light treatment, the plants were grown under low-light conditions (240 μmol m^−2^ s^−1^) and medium-light conditions (600 μmol m^−2^ s^−1^); control plants were grown in a field (high-light conditions, 1400–1600 μmol m^−2^ s^−1^, Figures S1E,F). For the temperature experiments, tea plants were grown at 15, 20, and 25°C with 75% humidity. The second leaves were collected for imaging and pigment assays.

### Photosynthetic pigment assays and ultra-structural observation

Photosynthetic pigment content was quantified as described previously (Arnon, 1949; Wei et al., 2012). The normal green tea cultivar *C. sinensis* (L.) O. Kuntze cv. *Rougui* was used as a control for pigment and ultra-structural analyses. For Chl analysis, we first performed a quick measurement of the relative amount of Chl content using a SPAD-502PLUS Chl meter (Spectrum Technologies, Konica Minolta, Japan) according to a previous report (Akita et al., 2013). Then, total Chl and carotenoids were extracted and quantified using a TU-1810PC spectrophotometer (Purkinje General Instrument Co. Ltd., Beijing, China). Lutein content was measured according to a previous report (Pan et al., 2007), and further confirmed by comparison with an authentic compound (Sigma-Aldrich, St. Louis, MO, USA) using an E2695 HPLC system (Waters, USA). The assays were conducted in triplicate.

Samples for ultra-structural observation were prepared as described previously (Du et al., 2008). Fresh tea leaves were cut into small pieces (1.0 × 2.0 mm) with a scalpel and fixed in glutaraldehyde solution (2.5%, v/v) overnight at 4°C. Ultrathin sections (70–90 nm) were prepared using a LKB Nova Ultra-microtome (LKB, Bromma, Sweden), examined and imaged using a JEM2100HC transmission electron microscope (JEOL, Tokyo, Japan).

### Measurement of antioxidant enzymes

Fresh leaves (0.5 g) were ground into a fine powder in liquid nitrogen, and then 4.5 mL phosphate-buffered saline (pH 7.4, 0.1 M) was then added to the leaves. The mixture was stirred on ice for 10 min and centrifuged at 5000 rpm for 10 min at 4°C. The supernatant was collected for enzyme activity assays. Superoxide dismutase (SOD), catalase (CAT), and peroxidase (POD) were assayed using commercial kits (Nanjing Jiancheng Bioengineering Institute, Nanjing, China) and a TU-1810PC UV-visible spectrophotometer (Persee, Beijing, China) according to the manufacturers' instructions and a previous report (Li H. X. et al., 2013).

### RNA isolation, library construction, and RNA-Seq

For Illumina HiSeq, total RNA was isolated from each sample using TRlzol reagent (Invitrogen™ Life Technologies, CA, USA) according to the manufacturer's recommendations and then purified using a Qubit® RNA Assay Kit with a Qubit® 2.0 Fluorometer (Life Technologies, CA, USA). RNA integrity was assessed using a 2100 Bio analyzer system (Agilent Technologies, CA, USA), with a minimum integrity value of 8.1. RNA degradation and contamination were monitored by 1% agarose gel electrophoresis. For RNA-Seq and DGE analyses, two biological replicates were used. Samples were harvested after the plants were shaded for 3 days (designated T3d_Z_1 and T3d_Z_2, T3d_W_1 and T3d_W_2) or 6 days (designated T6d_Z_1 and T6d_Z_2, T6d_W_1 and T6d_W_2). Total RNA (3 μg) per sample was pooled for 100-bp paired-end transcriptome sequencing. Four-microliter aliquots of each of the eight samples were used for expression profile analysis. cDNA library construction and Illumina sequencing were performed by Novogene Bioinformatics Technologies Co., Ltd. (Beijing, China).

Sequencing libraries were generated using a NEBNext Ultra RNA Library Prep Kit for Illumina® (NEB, USA), and index codes were added to attribute sequences to each sample. Briefly, mRNA was purified from 3 μg total RNA using poly-T oligo-attached magnetic beads. Fragmentation was performed using divalent cations under elevated temperature in NEBNext First Strand Synthesis Reaction Buffer (5X). First-strand cDNA was synthesized using random hexamer primers, and second-strand cDNA was subsequently synthesized. Double-stranded cDNA was amplified by PCR with Phusion High-Fidelity DNA polymerase, Universal PCR primers and Index (X) Primer. The PCR products were purified (AMPure XP system), and the library quality was assessed using an Agilent Bio analyzer 2100 system. The library preparations were sequenced from both the 5′ and 3′ ends using the Illumina HiSeq™ 2000 platform, and 100-bp paired-end reads were generated.

### Data preprocessing, *de novo* assembly and functional annotation

Raw sequencing reads were evaluated, and a unigene library was generated for *C. sinensis*. Raw data were preprocessed using in-house Perl scripts. Adaptor sequences, duplicated sequences, poly-N reads containing more than 10% “N,” and low-quality reads containing more than 50% bases with *Q* ≤ 5 were removed to obtain high-quality clean reads. After low-quality and ambiguous nucleotides were trimmed, *de novo* assemblies were created from the clean reads using Trinity (Grabherr et al., 2011). The data generated from the mixed samples were used to construct a whole transcriptome, which was used as a reference for further gene expression analysis.

For functional gene annotations, all non-redundant transcripts (≥ 200 bp) were used to search against public databases, including the National Center for Biotechnology Information (NCBI) non-redundant protein (Nr) and nucleotide (Nt) collections, Protein family (Pfam), Swiss-Prot, EuKaryotic Orthologous Groups (KOG), and Kyoto Encyclopedia of Genes and Genomes (KEGG) databases, with a significance threshold of *E* ≤ 10^−5^. For GO functional annotation analysis, GO terms were annotated according to molecular function, biological process and cellular component ontologies using Blast2GO software (Gotz et al., 2008).

### Gene expression analysis

For DGE analysis, the unigene dataset generated from the *C. sinensis* cv. *Baijiguan* transcriptome was used as a reference database. Clean reads were mapped to the reference sequences, and annotation information was obtained for each sample using RSEM software (Li and Dewey, 2011). To provide a relative assessment of transcript abundance, the fragments per kilobase of exon model per million mapped reads (FPKM) value was used as a measure of normalized gene expression (Mortazavi et al., 2008; Jakhesara et al., 2013). For samples with biological replicates, DEGs were identified with the DESeq R package using |log_2_(fold change)| ≥ 1 and a corrected *P* < 0.01 as the threshold for significant differential expression (Li C. et al., 2013; Sun et al., 2014). *P*-values were adjusted as described previously by Benjamini and Hochberg (1995).

GO enrichment analysis of all DEGs was implemented using the GOseq R package based on Wallenius non-central hyper-geometric distribution (Young et al., 2010). GO terms were assigned to the up- and down-regulated DEGs, with a corrected *P* = 0.01. For pathway enrichment analysis, all DEGs were mapped to pathways in the KEGG database using KOBAS software to identify significantly enriched KEGG pathways (Mao et al., 2005; Kanehisa et al., 2008). DEGs were considered significantly enriched in a metabolic pathway at a *q* ≤ 0.05 compared with the whole transcriptome background (Kanehisa et al., 2006).

### Validation of the DEGs by qRT-PCR

To validate the results of RNA-Seq and DGE analyses, 20 DEGs associated with the light-harvesting complex, photosynthetic pigment, flavonoid biosynthesis and peroxisome pathways were selected for qRT-PCR. Specific primers were designed using Primer 5.0; the primer pair sequences are listed in Table S1. First-strand cDNAs (10-fold dilution) synthesized from RNA extracted from the second leaves were used as templates. qRT-PCR was performed using a SYBR Premix Ex Taq Kit (TaKaRa, Dalian, China) and an Eco™ Real-Time PCR System (Illumina, USA) according to the manufacturers' instructions. The glyceraldehyde 3-phosphate dehydrogenase (*GAPDH*) gene was utilized as a loading control, and diethylpyrocarbonate (DEPC)-treated water in place of the template served as a negative control. PCR reaction efficiency was assayed for each primer set using a 10-fold dilution of cDNA. Each reaction was performed in triplicate along with an internal control reaction. Relative gene expression levels were calculated according to the 2^−Δ*ΔCt*^ comparative CT method (Livak and Schmittgen, 2001). The qRT-PCR and DGE analysis results are presented as fold changes in gene expression relative to the control samples. Therefore, the relative values of control samples were 1, and the relative values of the T3d_Z and T6d_Z samples were normalized to those of the control samples (T3d_W and T6d_W).

### Statistical analysis

*T-test* was performed using the SPSS 17.0 program (SPSS Inc., Chicago, USA). The letters following the values listed in the tables indicate significant differences (capital letters represent *P* < 0.05, and lower case letters represent *P* < 0.01). Significant differences are indicated with either one asterisk (*P* < 0.05) or two asterisks (*P* < 0.01) in the figures. All data are presented as the means ± standard error (SE, *n* = 3).

## Results

### Phenotypic and physiological characterization of a Chl-deficient yellow leaf variety

#### Flush shoots from Baijiguan exhibit a yellow phenotype and reduced Chl content under high light intensity

**Figure 1:**
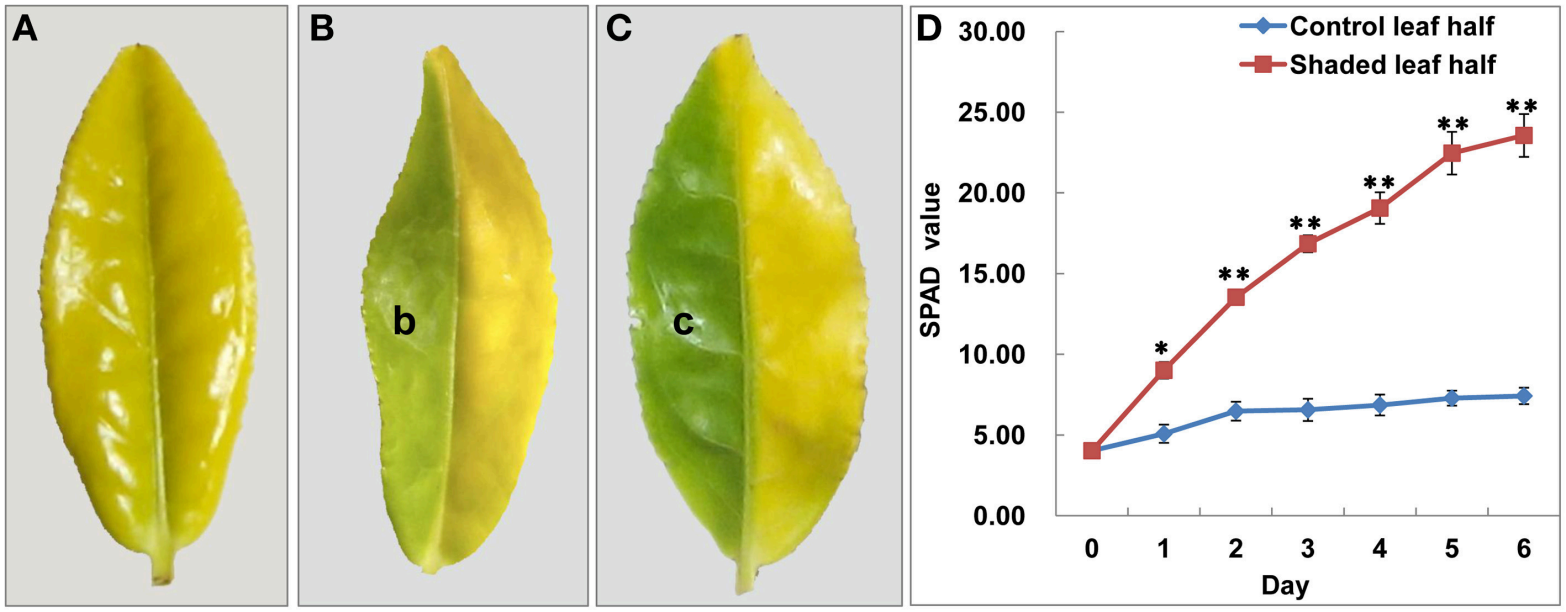
**Shade treatment affected the leaf phenotype and Chl content in *Baijiguan*. (A)** Phenotype of *Baijiguan* grown under field conditions. **(B)** Phenotype after 3 days of shading (b sector). The pale green leaf half was designated T3d_Z, and the control was designated T3d_W. **(C)** Phenotype after 6 days of shading (c sector). The green leaf half was designated T6d_Z, and the control was designated T6d_W. **(D)** Changes of relative chlorophyll content during the 6 days of shade treatment. All data points are presented as the means ± SE (*n* = 3).

*Baijiguan* plants showed an unusual leaf phenotype. In early spring, the first flush shoots were yellowish compared with a normal green tea cultivar (Figure 1A). In late spring, leaves of the *Baijiguan* plants retained a yellow phenotype at the one-bud and three-leaf stages, whereas leaves of most cultivars were dark green in color. On young shoots at the one-bud and four-leaf stages, the fourth leaf from the previous season gradually turned green, whereas the flush shoots remained yellow as the temperature and light intensity increased during the season. This phenomenon has also been reported for *Huangjinya* variety (Wang K. R. et al., 2013; Feng et al., 2014).

To characterize the leaf color phenotype of *Baijiguan*, we compared its pigment content with that of *Rougui*, a normal green cultivar (Table S2). Under high light intensity conditions, the total Chl, Chl a, Chl b, and carotenoid contents of *Baijiguan* were significantly lower than those of *Rougui*; however, the ratios of Chl a/Chl b and carotenoid/total Chl in *Baijiguan* were higher than those in *Rougui*.

#### Yellow leaf development in Baijiguan was dependent on the environmental light intensity

To test whether the yellow shoot phenotype of the *Baijiguan* variety is dependent on environmental factors, we grew the plants under different light and temperature conditions. Under low-light conditions, the leaf color of *Baijiguan* did not differ from that of the normal green cultivar. However, *Baijiguan* displayed green-yellow and yellow shoots under medium- and high- light intensity conditions, respectively (Figures S1A-F). The total Chl and carotenoid content also showed a decreasing trend as the light intensity increased (Figure S1G). In contrast, the yellow phenotype of *Baijiguan* was not affected when the plants were grown at different temperatures (15, 20, and 25°C).

To further confirm that the yellow leaf phenotype may result from exposure to high light intensity conditions, we conducted a light-shading experiment and observed that the sunlight-shielded portion of the leaf turned green, whereas the control portion remained yellow (Figures 1B,C). These observations demonstrated that *Baijiguan* is a light-hypersensitive variety.

Under shade conditions, the leaves of *Baijiguan* rapidly turned green, coinciding with the biosynthesis of Chl a, Chl b, and carotenoids (Table 1). The SPAD value, which reflects the visual perception of color differences, also increased by ~Six-fold in the shaded leaf half compared with the control half (Figure 1D). In contrast, the lutein content significantly decreased after 3 and 6 days of shade treatment (Table 1). These results indicated that the yellow phenotype in *Baijiguan* resulted from pigment composition changes.

**Table 1:** **Pigment content in *Baijiguan* under shade conditions**.

**Lable**	**Chl a (μg•g−1)**	**Chl b (μg•g−1)**	**Chl a/Chl b**	**Total Chl (μg•g−1)**	**Carotenoid (μg•g−1)**	**Carotenoid/Total Chl**	**Lutein (μg•g−1)**
T3d_W	119.78 ± 5.23a	24.11 ± 1.25a	3.52a	108.89 ± 3.58a	118.63 ± 2.26a	1.09a	86.44 ± 1.78a
T3d_Z	269.16 ± 7.03b	104.13 ± 4.10b	2.58b	373.29 ± 7.66b	99.27 ± 1.25b	0.27b	70.39 ± 2.96b
T6d_W	138.24 ± 5.83a	41.48 ± 2.38a	3.33a	179.72 ± 5.82a	128.95 ± 5.73a	0.72a	84.66 ± 2.46a
T6d_Z	345.36 ± 8.29b	139.87 ± 6.33b	2.47b	485.23 ± 9.23b	105.16 ± 3.04b	0.22b	49.37 ± 3.33b

*Data with different letters (a, b) in the same column represent significance at the level of P < 0.01. T3d_Z compared to T3d_W, and T6d_Z compared to T6d_W*.

#### Chloroplast structural changes in response to shading

**Figure 2:**
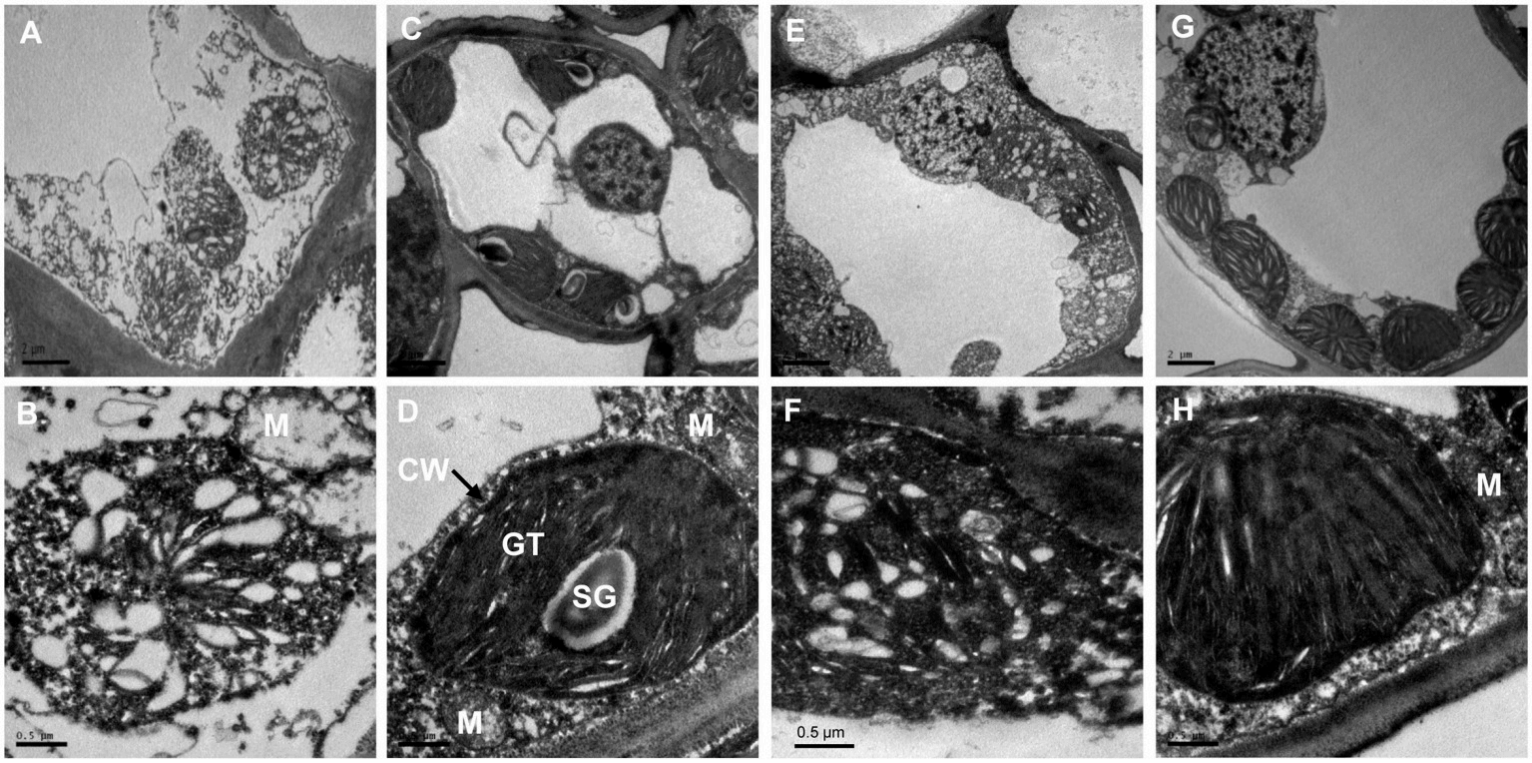
**Chloroplast ultra-structural comparison between *Baijiguan* and *Rougui* under high-light and shade conditions. (A,B)** Chloroplast ultra-structures in *Baijiguan* under high-light conditions. **(C,D)** Chloroplast ultra-structures in *Rougui* under high-light conditions. Chloroplasts in *Rougui* exhibit typical stacks and abundant starch granules compared with those in *Baijiguan*. **(E,F)** Chloroplast ultra-structures in the non-shaded part of *Baijiguan* at 6 days of shading. **(G,H)** Chloroplast ultra-structures in the shaded part of *Baijiguan* at 6 days of shading. Bar = 2 μm in **(A)**, **(C)**, **(E)**, and **(G);** 0.5 μm in **(B)**, **(D)**, **(F)**, and **(H)**. SG: starch granule, GT: grana thylakoid, M: mitochondria, and CW: cell wall.

To better understand the structural basis of the yellow phenotype, the leaf ultra-structures of *C. sinensis* cv. *Baijiguan* and *Rougui* were compared (Figure 2). Under high-intensity light, chloroplasts in *Rougui* developed a typical membrane system, with grana connected by stroma lamellae (Figures 2C,D). However, the grana stacks were less dense in *Baijiguan* compared with those in *Rougui*, and the thylakoid membrane system and spacing were disrupted (Figures 2A,B). Thus, the *Baijiguan* cultivar showed defects in chloroplast development.

Under shade conditions, the chloroplast ultra-structure of the control leaf half showed disordered grana stacking, similar to that of the yellow leaf under high light conditions (Figures 2E,F). After 6 days of shading, the chloroplasts exhibited stacked thylakoids that were distributed around the cell wall similar to common green cultivars, except that fewer starch grains were found in *Baijiguan* (Figures 2G,H). These results indicated that the yellow phenotype in *Baijiguan* was induced by high light intensity and could be rescued by reducing the light intensity.

#### Antioxidant enzyme activities in *C. sinensis* cv. Baijiguan increased after shading

**Figure 3:**
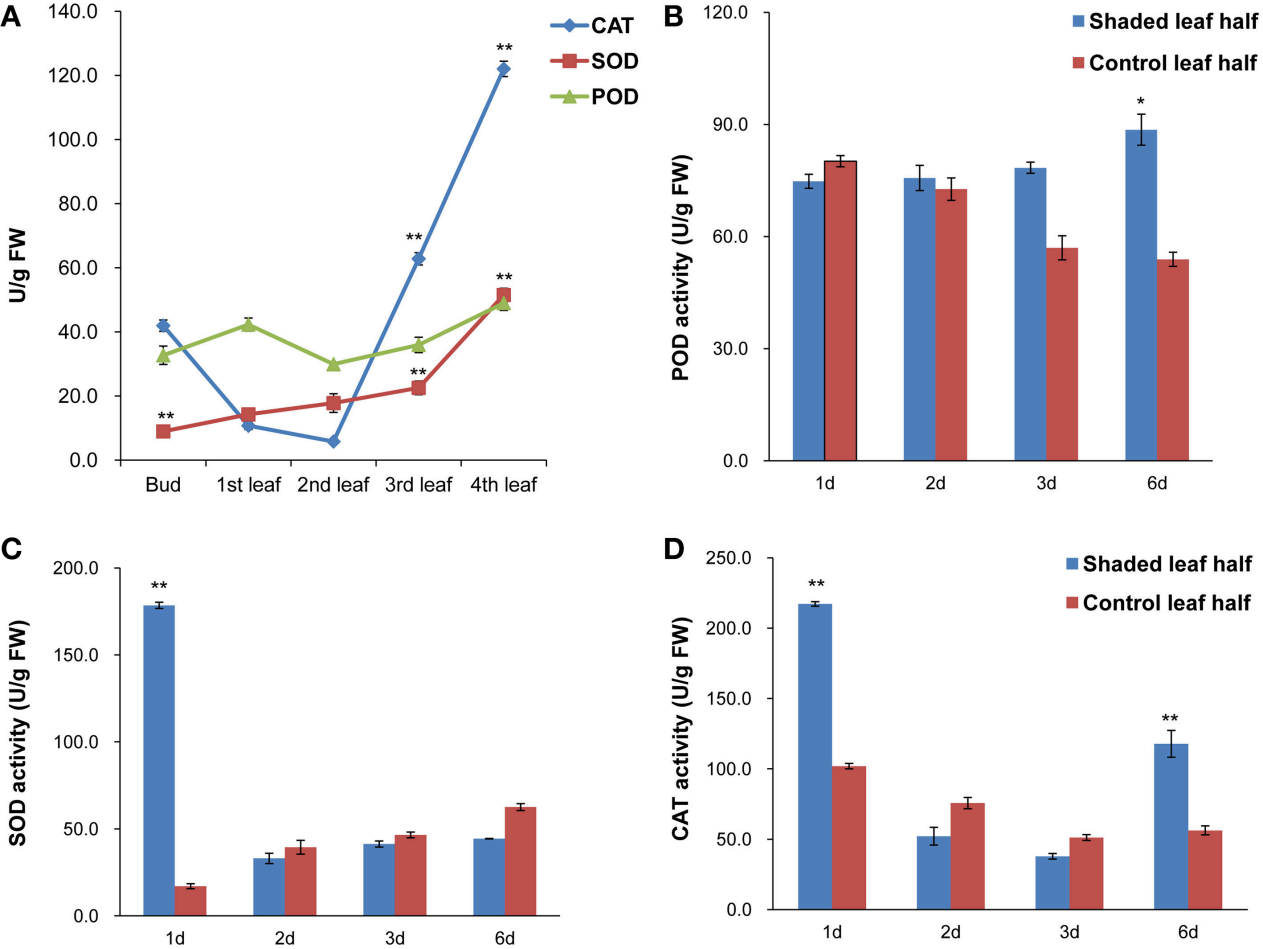
**Antioxidant enzyme activities in *C. sinensis* cv. *Baijiguan*. (A)** Antioxidant enzyme activities at different leaf positions under high-light conditions. The second leaf was utilized as the control. **(B)** Effects of shading on POD activity. **(C)** Effects of shading on SOD activity. **(D)** Effects of shading on CAT activity. ^*^ Represents a significant difference (*P* < 0.05) between the control and shade treatment. ^**^ Represents a highly significant difference (*P* < 0.01) between the control and shade treatment. In Panel **(A)**, other leaf position compared to the second leaf position, and in Panels **(B–D)**, T3d_Z compared to T3d_W and T6d_Z compared to T6d_W.

Antioxidant enzyme activities of SOD, CAT and POD at different leaf positions under high light conditions and in the second leaf under shade treatments were analyzed. Under high light conditions, SOD and CAT activities in the third and fourth leaves were higher than in the second leaf, whereas POD activity showed no significant changes. Overall, the antioxidant enzyme activities in the second leaf were the lowest compared with the other analyzed leaf positions (Figure 3A). Under shade treatment, SOD activity increased significantly, by ~10-fold compared to the control after 1 day of shading, and CAT activity was 2 times higher than the control value (Figures 3C,D). The activities of both SOD and CAT activities were decreased after 2 and 3 days of shading. Following 6 days of shading, slightly higher POD activity was observed (Figure 3B). These results indicated that the second leaf of *Baijiguan* might experience much higher oxidative stress under high light intensity conditions.

### Transcriptome analysis

#### *De novo* transcriptome sequencing, assembly, and blast analysis

To obtain an overview of transcriptome remodeling during shade treatments, a cDNA library was constructed using equal amounts of RNA from the second leaves of plants after 3 days (Figure 1B) and 6 days (Figure 1C) of shading. In total, we obtained 16.1 Gb of clean data (accounting for 97.34% of the raw data) from paired-end reads, with a single read length of ~100 bp and a Q20 percentage of over 97%. These clean reads were assembled into 88,788 unigenes representing 170,201 assembled unique transcripts, with an average length of 641 bp and an N50 length of 1021 bp (Table 2). Among these unigenes, 29,856 genes are longer than 500 bp and accounted for 33.63% of all unigenes. Additionally, 14,547 genes are longer than 1.0 kb and accounted for 16.38% of all unigenes. This dataset has been deposited at NCBI Short Read Archive (SRA) (accession number: SRX1078570).

**Table 2:** **Assembly results of the *C. sinensis* transcriptome using Trinity software**.

**Unigene length (bp)**	**Number of unigenes**	**Percentage**
200–500	58,932	66.37
500–1000	15,309	17.24
1000–2000	9219	10.38
2000+	5328	6.00
Total number	88,788	
Mean length	641	
N50 length	1021	

To predict the functions of the assembled transcripts, the 88,788 unigenes were annotated by searching the Nr, Nt, Pfam, KOG, Swiss-Prot, GO, and KEGG public databases (Table 3). In total, transcriptome sequencing of all eight samples resulted in the annotation of 35,703 unigenes, accounting for 40.21% of all unigenes. Among these unigenes, 34.97% were annotated to the Nr databases. Of the Nr annotated unigenes, 46.67% had close homology to *Vitis vinifera*, followed by *Populus trichocarpa* and *Ricinus communis* (Figure S2).

**Table 3:** **Summary of annotations of the *C. sinensis* transcriptome**.

**Annotation database**	**Number of Unigenes**	**Percentage**
Nr	31,058	34.97
Nt	15,354	17.29
KO	8561	9.64
Swiss-Prot	20,797	23.42
PFAM	21,869	24.63
GO	24,846	27.98
KOG	10,213	11.50
Unigenes hit all seven databases	2228	2.50
Unigenes hit at least one Database	35,703	40.21
Go annotations for Arabidopsis protein hits	5565	6.27
Total Unigenes	88,788	100

#### Functional annotation and pathway assignment

GO analysis resulted in the classification of 24,846 unigenes into 47 sub-categories (Figure S3). Within the biological process category, “cellular process” (14,960 unigenes, 60.21%), and “metabolic process” (13,949 unigenes, 56.14%) were the top two GO terms. The most highly represented molecular function terms were “binding” (14,244 unigenes, 57.33%), “catalytic activity” (11,937 unigenes, 48.04%), and “transporter activity” (1773 unigenes, 7.14%). The top three GO terms in the cellular component category included “cell” (8975 unigenes, 36.12%), “cell part” (8946 unigenes, 13.00%), and “organelle” (6366 unigenes, 25.62%).

A total of 10,213 unigenes were subdivided into 26 groups according to the KOG database (Figure S4). Among these classifications, the “general functional prediction only” cluster (1984 unigenes, 19.43%) represented the largest group, followed by “post-translational modification, protein turnover, chaperone” (1345 unigenes, 13.17%), “signal transduction” (870 unigenes, 8.52%), and “translation” (705 unigenes, 6.90%). In addition, “lipid metabolism” and “cell wall/membrane/envelope biogenesis,” which are closely related to cell structure, were annotated.

KEGG provides a network diagram of cell metabolic pathways. A total of 8561 unigenes were assigned to specific pathways (Figure S5). The top enriched pathways were “carbohydrate metabolism” (792 unigenes), “translation” (772 unigenes), “folding, sorting and degradation” (640 unigenes), “signal transduction” (626 unigenes), and “energy metabolism” (621 unigenes).

#### Identification and categorization of TFs

**Figure 4:**
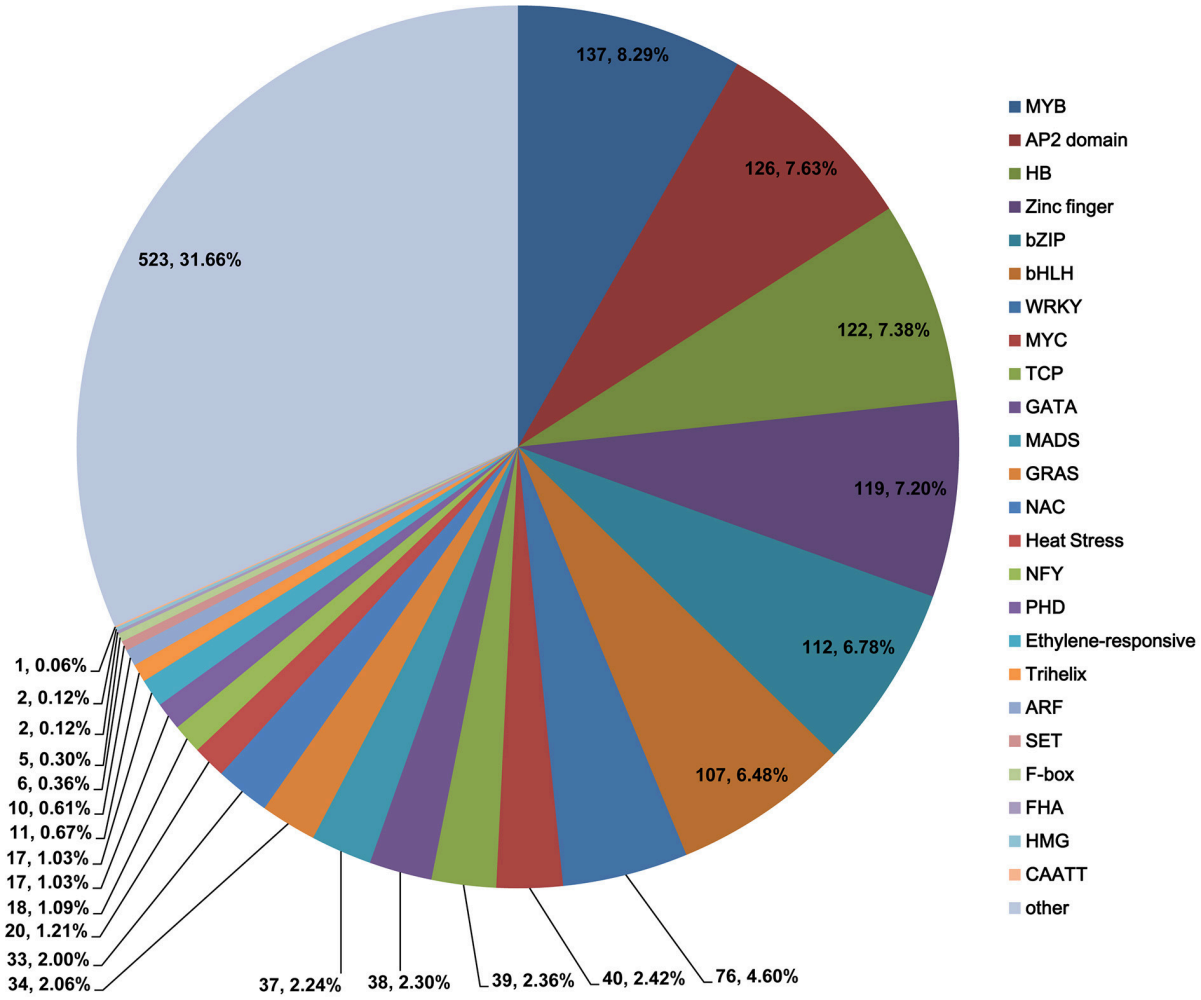
**Numbers and percentages of unique transcription factors identified in this study**.

TFs play significant roles in plant development and response to environmental stimuli. In this study, 1652 TFs were identified and categorized into 25 different common families (Figure 4). The MYB TF family was the most predominant (137 TFs, 8.29%), followed by the AP2 domain (126 TFs, 7.63%), homeobox (HB, 122 TFs, 7.38%), zinc finger (119 TFs, 7.20%), bZIP (112 TFs, 6.78%), and bHLH (107 TFs, 6.48%) families. The identification of these TFs provides opportunities for understanding their functions in light-regulated chloroplast development.

### Comparison of unigenes differentially expressed under shade conditions

#### DGE library sequencing and mapping

DGE analysis of T3d_Z, T3d_W, T6d_Z, and T6d_W was performed using an Illumina HiSeq™ 2000 sequencing platform with two biological replicates; the Pearson correlation coefficients between these two replicates ranged from 0.913 to 0.926 (Robles et al., 2012; Herzel and Neugebauer, 2015). A total of 1.59 Gb to 2.1 Gb of clean data was obtained, accounting for 99.7% of the raw data for each sample. The clean reads were mapped to reference sequences derived from the *C. sinensis* transcriptome data. The total proportion of mapped reads ranged from 91.05 to 92.42% (Table S3).

#### Genes differentially expressed at the same time points after shading

**Figure 5:**
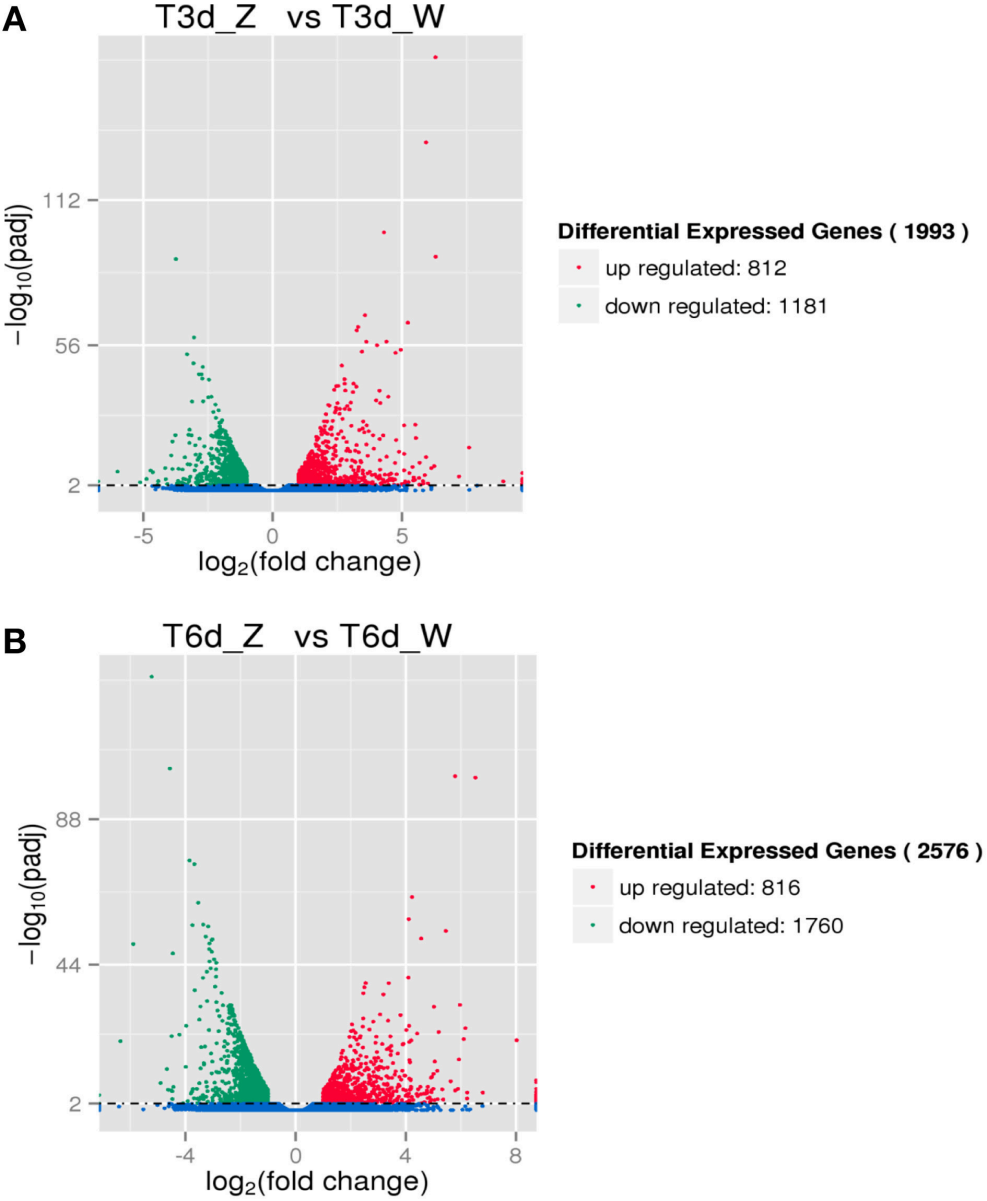
**DEGs due to shade treatment. (A)** Differentially expressed transcripts after 3 days of shading. **(B)** Differentially expressed transcripts after 6 days of shading.

Two-group analysis was performed to identify *C. sinensis* genes differentially expressed after three and 6 days of shade treatment (Figure 5). The number of down-regulated genes exceeded the number of up-regulated genes following this treatment. As the shading time increased, the number of DEGs in *C. sinensis* gradually increased from 1993 to 2576. After 6 days of shading, a 1.29-fold increase in the number of induced DEGs was observed compared with that detected after 3 days of shading. Specifically, the up- and down-regulated genes increased in number by 4 and 579, respectively.

#### Differentially co-expressed genes at different time points after shading

**Figure 6:**
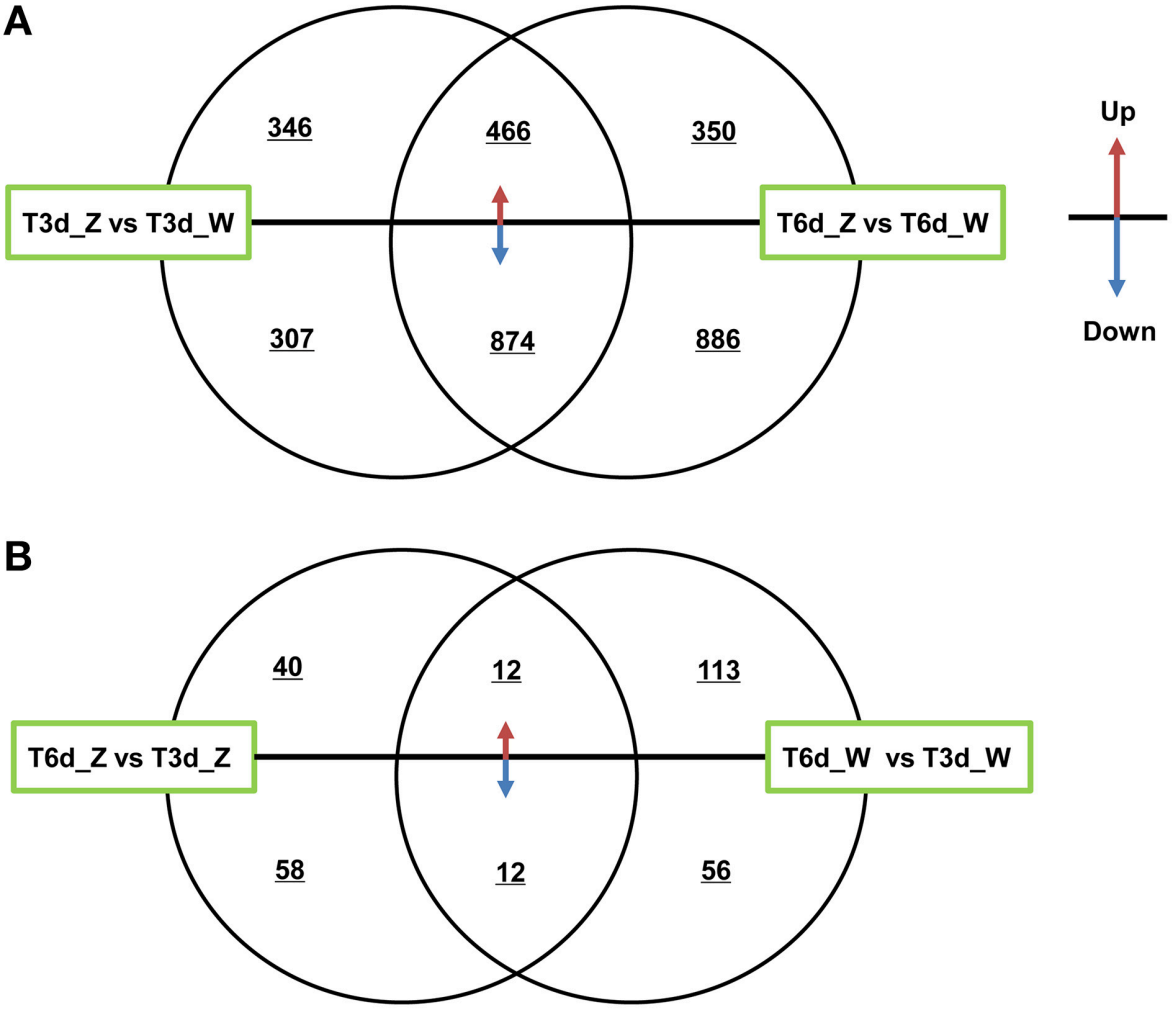
**Differentially co-expressed genes at different time points after shading**. **(A)** A comparison between the T3d_Z vs. T3d_W and T6d_Z vs. T6d_W groups. **(B)** A comparison between the T6d_Z vs. T3d_Z and T6d_W vs. T3d_W groups. The up or down arrows revealed that DEGs were up- or down-regulated.

The two shading treatment groups were subjected to multi-group differential expression analysis. The differentially co-expressed genes in the treatment and control groups are identified and illustrated in the Venn diagram shown in Figure 6. A total of 653 unique DEGs were identified in the T3d_Z vs. T3d_W group (346 up- and 307 down-regulated DEGs, Figure 6A), and 1236 unique DEGs were identified in the T6d_Z vs. T6d_W group (350 up- and 886 down-regulated). A comparison of the differentially co-expressed genes between the T6d_Z vs. T6d_W and T3d_Z vs. T3d_W groups resulted in the identification of 1340 DEGs (466 up- and 874 down-regulated DEGs, Table S4). Considering that the leaf color became greener with increased shading time (Figures 1B,C), these differentially co-expressed genes in the two shade-treated groups may play important roles in light-responsive regulation, leaf color formation and metabolic networks.

Among the DEGs between the control and shade-treated groups, including both the T3d_Z vs. T3d_W and T6d_Z vs. T6d_W groups, 81 genes encoding putative TFs in *C. sinensis* were identified. Most of these TFs were classified into the bHLH (11), MYB (10), zinc finger (6), AP2 domain (5), bZIP (3), and homeobox (3) families (Table S5). MYB and bHLH TFs have crucial roles in secondary metabolism (particularly flavonoid biosynthesis) and abiotic stress (Roig-Villanova et al., 2007; Zhao et al., 2012; Wang et al., 2015).

The DEGs in the control and treatment groups were analyzed, and 98 unique DEGs were identified in the T6d_Z vs. T3d_Z group (40 up- and 58 down-regulated DEGs, Figure 6B). These data provide clear evidence that the genes identified as up-regulated are associated with oxidoreductase activity and that the down-regulated genes are associated with the light-harvesting complex and Chl A/B binding protein. These genes can be considered as candidate light-responsive genes in future studies (Table S6).

### Go classification of DEGs

#### Categories of down-regulated genes revealed by GO analysis

A comparison of the 3-day shaded samples with the control revealed 1993 DEGs, including 1181 down-regulated and 812 up-regulated genes, which were assigned to relevant functional terms. The top significantly enriched GO terms in the biological process category were “metabolic process,” “single-organism metabolic process,” and “biosynthetic process”; those in the cellular component category were “intracellular organelle” and “organelle”; and that in the molecular function category was “oxidoreductase activity” (Figure S6A). A comparison of the 6-day shaded samples with the control revealed 2576 DEGs, including 1760 down-regulated and 816 up-regulated genes; these DEGs were assigned to relevant functional terms. The results for the biological process and molecular function categories were similar to those of the above comparison, whereas “cytoplasm” and “cytoplasmic part” were significantly overrepresented in the cellular component category (Figure S6B). Overall, these data suggested that the terms “metabolic process,” “oxidoreductase activity,” “cytoplasm,” and “organelle” are strongly affected by shading. Other down-regulated GO terms, including “plastid,” “chloroplast,” and “thylakoid,” were highly enriched among the DEGs, supporting the efficiency of the light treatments and the reliability of the gene expression data.

#### Categories of up-regulated genes

All of the GO terms assigned to the group of up-regulated genes converged into two main categories (Figures S6C,D): “oxidation-reduction processes” in biological process and “oxidoreductase activity” in molecular function. Therefore, yellow leaves are highly susceptible to oxidative damage.

### Analysis of DEGs involved in the light-responsive network and leaf color formation

**Figure 7:**
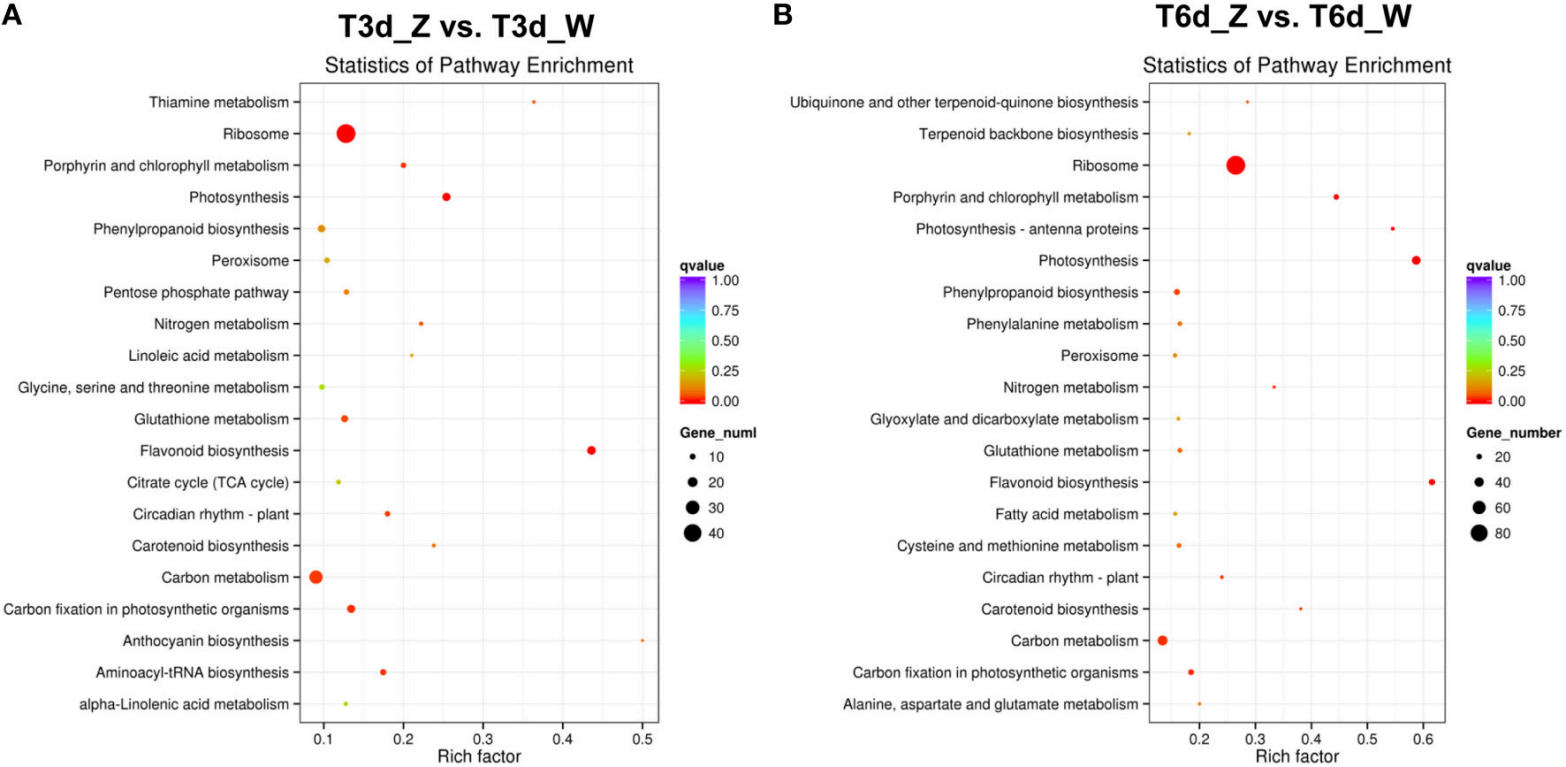
**Top 20 enriched KEGG pathways among the annotated DEGs across two comparisons. (A)** T3d_Z vs. control T3d_W; and **(B)** T6d_Z vs. control T6d_W. The Y-axis on the left represents KEGG pathways, and the X-axis indicates the enrichment factor. Low *q*-values are shown in red, and high *q*-values are depicted in blue.

To determine whether light-responsive genes were enriched, the KEGG pathway database was searched using the DEGs to reveal the top 20 significantly enriched pathways (Figure 7 and Table S7). The following KEGG pathways were enriched after the 3-day shade treatment: 43 DEGs were enriched in “ribosome,” 29 DEGs were related to “carbon metabolism,” and 17 DEGs were related to “flavonoid biosynthesis” (Figure 7A), corresponding to ~12.8, 13.4, and 43.6% of the total DEGs, respectively. More than 20% of the DEGs were found to be associated with the “photosynthesis,” “porphyrin and Chl metabolism,” and “carotenoid biosynthesis” pathways, suggesting that low-light stress affects plant photosynthesis (Ho et al., 2009; Li C. et al., 2013). Similar results were observed for the 6-day shaded sample, in which more than 38.0% of the genes were enriched for “photosynthesis,” “porphyrin and Chl metabolism,” and “carotenoid biosynthesis” (Figure 7B). In addition, the “nitrogen metabolism,” “phenylpropanoid biosynthesis,” “glutathione metabolism,” “peroxisome,” and “circadian rhythm-plant” pathways were enriched in both groups, and these enrichments increased with time. Notably, 12 genes related to the light-harvesting complex were enriched in the “photosynthesis-antenna proteins” pathway in the 6-day shade group. Genes related to “plant hormone signal transduction,” “terpenoid backbone biosynthesis,” and “fatty acid degradation” were up-regulated, and these pathways were enriched in both the T3d_Z vs. T3d_W and T6d_Z vs. T6d_W groups (Figure S7), suggesting that they might be involved in the response to shading and in the formation of leaf color.

**Figure 8:**
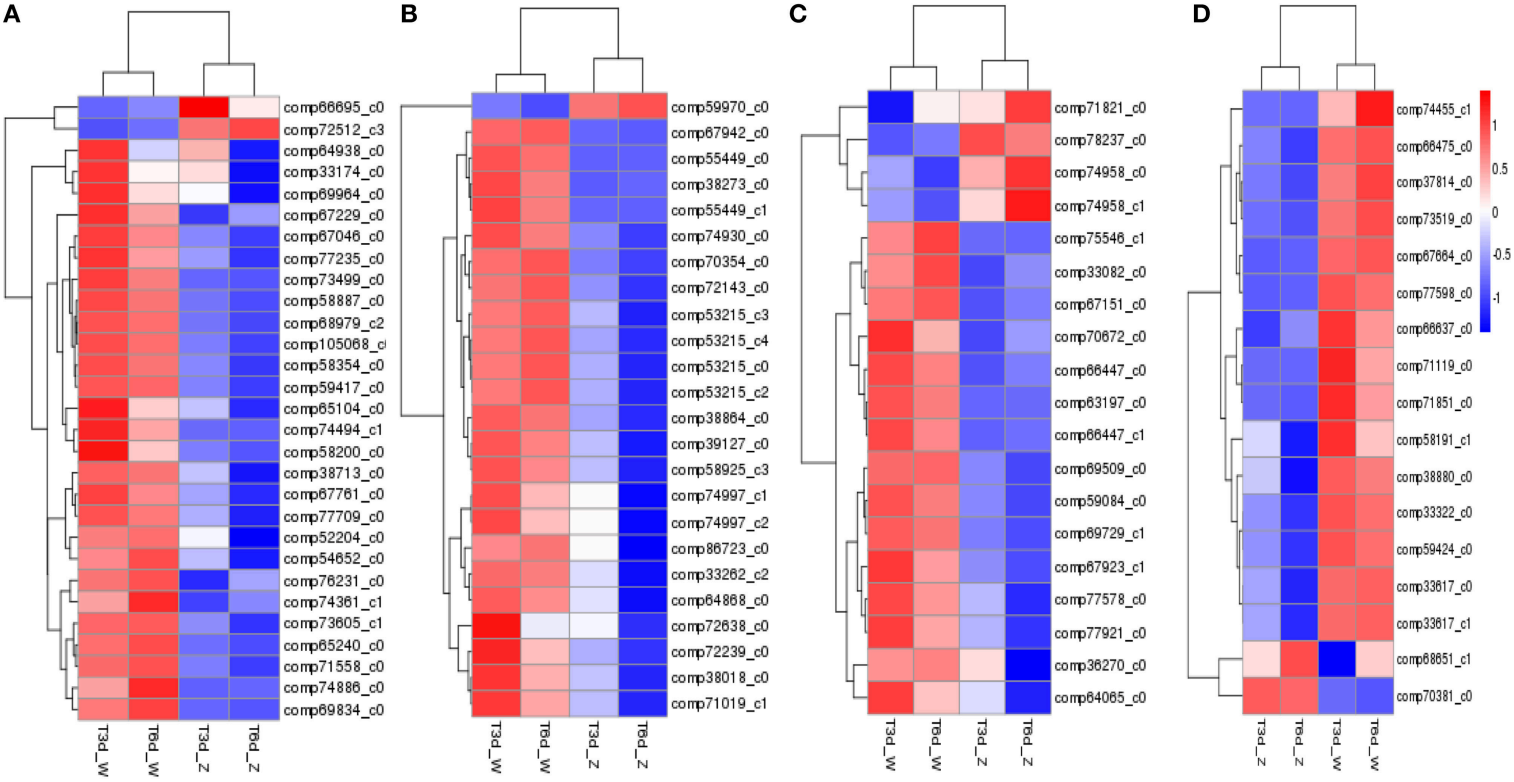
**Expression profile clustering at different time points. (A)** Photosynthetic pigment; **(B)** flavonoid biosynthesis; **(C)** glutathione metabolism; **(D)** peroxisome pathway. Expression ratios are expressed as log_10_ values, and each horizontal bar represents a single gene.

Clustering analysis and heat map results for four representative pathways (photosynthetic pigment, flavonoid biosynthesis, glutathione metabolism and peroxisome) indicated clustering of the two treatment groups and of the two control groups (Figure 8). Overall, the shaded leaf portions appeared to lack gene products compared with the non-shaded portions. Notably, among those DEGs, *POR* (comp66695_c0, fold change = 3.23 in the 3-day shade group), encoding the Chl metabolism enzym protochlorophyllide oxidoreductase, and *PsbR* (comp38841_c0, fold change = 5.22 in the 3-day shade group, fold change = 4.03 in 6-day shade group), encoding the photosynthesis-related photosystem II 10-kDa protein, were significantly up-regulated; other DEGs were down-regulated (Table S4).

In summary, the transcriptome data were consistent with the *Baijiguan* phenotype. The abnormal leaf color may result from changes in the expression of genes involved in pigment biosynthesis and reactive oxygen species (ROS) scavenging.

### qRT-PCR analysis of candidate genes involved in the light stress response

**Figure 9:**
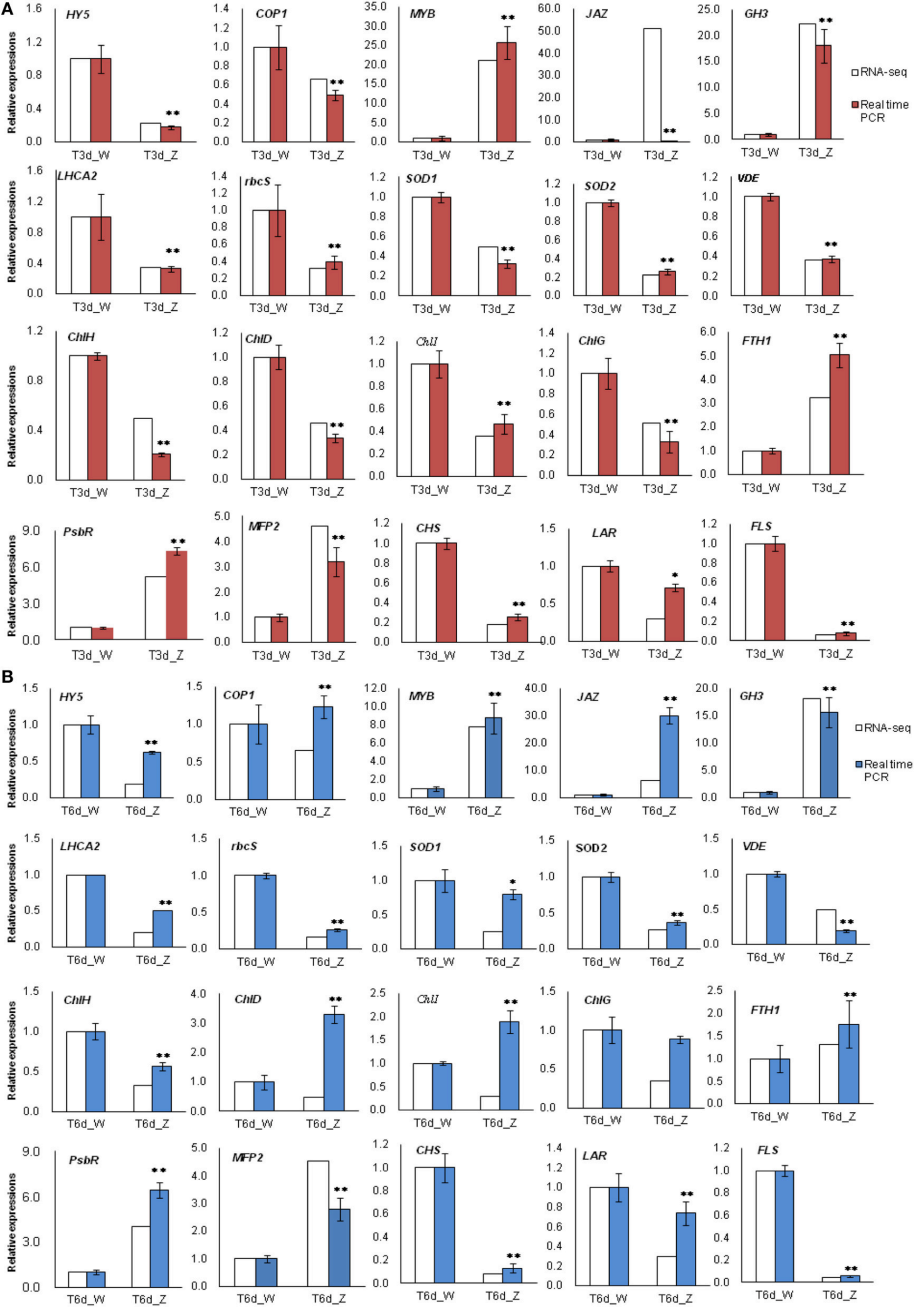
**Real-time PCR results confirmed differentially expressed transcripts identified by RNA-Seq**. Twenty genes involved in the light response were selected for validation. **(A)** T3d_Z compared to control T3d_W and **(B)** T6d_Z compared to control T6d_W. Data are normalized to *GAPDH* expression and are presented as the means ± SE (*n* = 3). ^*^ Represents a significant difference (*P* < 0.05) between the control and shade treatment. ^**^ Represents a highly significant difference (*P* < 0.01) between the control and shade treatment.

Among all the identified DEGs, those genes involved in the light-harvesting complex, and those genes related to porphyrin and Chl metabolism, carotenoid biosynthesis, flavonoid biosynthesis and peroxisome are closely related to the observed changes in leaf color during shade treatment. Twenty of these genes were selected for qRT-PCR analysis. In the 3-day shade group, the expression patterns of 19 genes, which were detected by qRT-PCR, were similar to those observed in the DGE data, whereas the *JAZ* gene showed a different expression pattern (Figure 9A). In the 6-day shade group, three genes (*COP1, ChlD*, and *ChlI*) showed different expression patterns compared with the DGE data (Figure 9B). In general, the qRT-PCR data were consistent with the Illumina sequencing results, suggesting that the RNA-Seq data are reliable.

## Discussion

The color variation of tea leaves has attracted much attention because the special chemical composition of these leaves affect tea quality. Light-shading experiments and ultra-structural observations have suggested that *Baijiguan* plants are hypersensitive to high-intensity light and have defects in Chl synthesis and chloroplast development. Therefore, this cultivar is an ideal system for studying the molecular mechanisms underlying leaf color formation.

### The yellow leaf phenotype is closely related to Chl pigment metabolism

The development of yellow leaves is related to pigment metabolism and chloroplast development. Leaf tissue has three major pigments: Chls, carotenoids and flavonoids. Chls are essential for photosynthesis, as these pigments are responsible for harvesting energy, charge separation and electron transport in antenna systems. Carotenoids, which are primarily synthesized in chloroplast membrane, form photosynthetic complexes and play critical roles in protecting Chl from destruction. Photosynthetic pigment measurements revealed that the yellow phenotype of *Baijiguan* positively correlated with the contents of Chls and carotenoids, particularly Chl a and Chl b (Table 1). Our results indicated that the yellow phenotype of *Baijiguan* might result from deficient Chl and carotenoid contents.

DEGs involved in carotenoid biosynthesis were identified based on the transcriptome and DGE data, and all 8 DEGs were down-regulated. One of these DEGs, *VDE*, which is involved in the xanthophyll cycle, which has a critical function in the photoprotection of photosystem II (Jahns and Holzwarth, 2012). Lutein is the most abundant xanthophyll and contributes to light harvesting by transferring excitation energy to Chl in higher plants. The lutein content was significantly decreased under shade conditions (Table 1). Moreover, the observed changes in the lutein content coincided with the *VDE* expression, thus suggesting that the xanthophyll cycle still functions normally under high-light intensity conditions and is not the cause for the yellow phenotype.

DEGs involved in Chl biosynthesis were also identified. Chl metabolism is a highly coordinated process that is catalyzed by numerous enzymes, particularly Mg-chelatase and protochlorophyllide reductase, via a series of cooperative reactions from glutamyl-tRNA to Chl a and Chl b (Zhang et al., 2006; Sakuraba et al., 2013). Mg-chelatase, which includes the subunits *ChlD, ChlH*, and *ChlI*, plays an important role in Chl production. Indeed, plants lacking Mg-chelatase show defective Chl and exhibit a yellow leaf phenotype (Jung et al., 2003). Thus, we examined the expression profiles of *CsChlH, CsChlD*, and *CsChlI* in the DGE data. Analysis of T3d_Z vs. T3d_W revealed that *CsChlH* (fold change = 0.49), *CsChlI* (fold change = 0.36), and *CsChlD* (fold change = 0.46) were down-regulated in *Baijiguan*. Analysis of T6d_Z vs. T6d_W showed that *CsChlH* (fold change = 0.33), *CsChlI* (fold change = 0.30), and *CsChlD* (fold change = 0.47) were also down-regulated. The expression levels of the three subunits of Mg-chelatase were not disrupted in *Baijiguan*; thus, they are not the causes of the yellow shoot phenotype. However, *POR* and *ChlP*, which encode protochlorophyllide oxidoreductase and a geranylgeranyl reductase, respectively, and which are essential for Chl synthesis under high light intensity conditions (Sakuraba et al., 2013; Zhou et al., 2013), were differentially expressed and identified in RNA-Seq data (comp66695_c0 and comp52204_c0). One study found that a light-induced yellow leaf mutant of rice is hypersensitive to high light intensity conditions and is defective in Chl synthesis. The light-induced leaf gene encoding geranylgeranyl reductase affects chlorophyll biosynthesis and light sensitivity (Zhou et al., 2013). However, we found that *ChlP* was down-regulated after shade treatment, suggesting that this gene may not be the cause for the yellow leaf development under high light intensity conditions. Compared with the non-shaded leaf, *POR* was significantly up-regulated after shading; Chl levels in an *Arabidopsis PORC* mutant have been shown to decrease dramatically under high light, and overexpression of *AtPORC* has been shown to increase the tolerance to photo-oxidative damage (Masuda et al., 2003; Pattanayak and Tripathy, 2011). These results suggest that *POR* was inhibited under high light intensity conditions, which might result in a reduced Chl content and the yellow-leaf phenotype.

### The yellow leaf phenotype positively correlates with PSII stability and the ROS scavenging system

Our light-shading experiments demonstrated that the *Baijiguan* cultivar displays increased sensitivity to high light intensity stress, which ROS and stimulates lipid peroxidation, resulting in the attacks on various cellular components (Li et al., 2015). In plants, ROS scavenging enzymes are induced in chloroplasts and mitochondria to detoxify ROS produced during abiotic stresses and to protect tissues from oxidative damage under stress conditions (Apel and Hirt, 2004; Cluis et al., 2004; Foyer and Noctor, 2005; Yoshimura et al., 2008). Genes encoding antioxidant enzymes, including superoxide dismutase (*SOD*, comp38880_c0, comp71851_c0), catalase (*CAT*, comp74455_c1), and glutathione peroxidase (*GSH-PX*, comp33082_c0, comp63197_c0), were identified among the DEGs (Figure 8D). Accordingly, we assayed the activities of SOD, CAT and POD. Among antioxidant enzymes, SOD plays a key role in cellular ROS detoxification. Our results showed that SOD activity increased markedly from 17.070 ± 1.42 to 178.52 ± 1.76 U/g FW after shade treatments (Figure 3). Therefore, this enzyme may be the first line of defense against the ROS that were generated in response to high light intensity stress, this enzyme clears harmful molecules such as O^2−^ to reduce photo-oxidative stress.

Transmission electron microscopy analysis revealed that the ultra-structure of chloroplasts in *Baijiguan* was disrupted under high light intensity conditions, exhibiting poorly stacked grana (Figures 2A,B). However, the yellow leaves of *Baijiguan* changed to green when the light intensity was reduced; this color change coincided restoration of the typical chloroplast structure and an increase in the Chl content. Strong sunlight appears to suppress grana stacking and thylakoid development, resulting in underdeveloped chloroplasts. These results were consistent with the observed changes in transcriptional abundance. GO annotation revealed the enrichment of many light- and chloroplast-related categories, including “light-harvesting complex,” “plastid,” “chloroplast,” and “thylakoid.” Eight DEGs (comp38841_c0, comp66018_c0, comp28142_c0, comp76361_c1, comp61823_c0, comp37286_c0, comp67545_c0 and comp11002_c0) related to chloroplast (GO: 0009507) were significantly up-regulated in both the T3d_Z vs. T3d_W and T6d_Z vs. T6d_W groups, suggesting crucial roles for these DEGs in repairing chloroplast structure. KEGG pathway enrichment analysis revealed that “fatty acid degradation” was up-regulated. The modification of membrane fluidity is mediated by fatty acid desaturation (Upchurch, 2008; Chen and Thelen, 2013). One gene encoding a chloroplast ω-6 fatty acid desaturase has been identified in *Arabidopsis thaliana*, and mutation of this gene results in newly developed leaves becoming yellow at low temperature (Hugly and Somerville, 1992). Other genes (*FAD3, FAD7* and *FAD8*) encoding ω-3 fatty-acid desaturases have been found to be differentially regulated in response to light (Collados et al., 2006). These results are consistent with the observations that non-shaded cells were devoid of thylakoid membranes and that light-shaded cells showed normal chloroplast structures (Figures 2E–H).

Pathway enrichment analysis revealed enrichment of both the “photosynthesis antenna proteins” and “photosynthesis” pathways. In the “photosynthesis antenna proteins” pathway, genes encoding light-harvesting complex I Chl a/b binding protein (*LHCA*) and light-harvesting complex II Chl a/b binding protein (*LHCB*) were down-regulated during shade treatment, suggesting that the yellow phenotype may not be related to the light-harvesting complex. However, *PsbR*, a 10-kDa photosystem II protein, was significantly up-regulated, whereas other genes involved in PSII stability were down-regulated after shade treatment (Figure 9 and Table S7). These results suggested that high light intensity inhibits *PsbR* expression in *Baijiguan*, thus affecting PSII stability and photosynthetic oxygen evolution (Suorsa et al., 2006; Liu et al., 2009). Up-regulation of the photosynthetic gene *PsbR* under shade conditions likely reduces photo-oxidative damage and restores chloroplast structure, ultimately turning the yellow leaves to a normal green color.

### TFs were positively induced by shading

TFs play critical roles in coordinating development with environmental changes. In this study, 1652 TFs were identified. Many of these TFs belong to the MYB, AP2 domain, homeobox, zinc finger, bZIP, and bHLH families, whose expression levels are primarily affected by abiotic stress (Obertello et al., 2010; Wang X. C. et al., 2013).

A total of 11 genes encoding bHLH TFs were identified in both the T3d_Z vs. T3d_W and T6d_Z vs. T6d_W groups (8 up- and 3 down-regulated genes, Table S5). It is reported that bHLH135 is involved in light signaling in *Arabidopsis* (Castelain et al., 2012); in addition, two genes encoding bHLH factors that were rapidly up-regulated after shading; these genes played important roles in integrating shade and hormone transcriptional networks (Roig-Villanova et al., 2007). The MYB family has also been implicated in the light-mediated regulation of plant development, stress responses, and pigment biosynthesis (Yamagishi et al., 2012). In our study, 10 MYB family genes were identified in the above groups (6 up- and 4 down-regulated genes, Table S5). Expression of a light-regulated MYB gene has been shown to be induced following exposure of etiolated or dark-adapted *Arabidopsis* seedlings to light (Quaedvlieg et al., 1996). In *Rehmannia glutinosa*, seven MYB genes were found to be up-regulated in the leaf or tuberous root under shade conditions (Wang et al., 2015). In *Litchi chinensis* Sonn, nine and five genes were up- and down-regulated, respectively, in response to shading (Li C. et al., 2013). Thus, MYB and bHLH family TFs are positively induced by shading and may play crucial roles in transcriptome reprogramming and leaf color formation.

### Circadian systems and phytohormone transcriptional signaling networks mediate light perception

Photoreceptors (phytochromes and cryptochromes) mediate light perception and regulate the transcription or activity of TFs via post-translational modifications (Deng et al., 2014). *PHYB* (phytochrome B, comp53675_c0), *CRY* (cryptochrome, comp59933_c0), *COP1* (constitutive photomorphogenic 1, comp74256_c0), *HY5* (long hypocotyl 5, comp38740_c0), and *LHY* (late elongated hypocotyl transcription factor, comp71228_c0) were among the identified DEGs (see Table S7 and Figure 9). A previous in *vitro* analysis revealed that the HY5 binds directly to the promoter of several light-inducible genes (Hiltbrunner et al., 2006).

*HY5* integrates the light and hormone signaling networks. A homolog gene of *HY5, STF1*, in *Glycine max* plays important roles in light and hormone signaling and in Chl accumulation (Song et al., 2014). *HY5* has also been reported to be involved in the light and jasmonic acid signaling pathways (Prasad et al., 2012). In the present study, the expression level of *HY5* was found to be down-regulated, whereas the expression levels of genes encoding auxin-responsive *GH3* (comp72299_c0) and jasmonate ZIM domain-containing protein (*JAZ*, comp36449_c0) were significantly up-regulated under shade stress (Figure 9). Our data support the notion that *HY5* promotes the expression of negative regulators of auxin signaling (Cluis et al., 2004), suggesting that the shade response of *C. sinensis* is also associated with jasmonic acid and auxin signaling networks.

### Secondary metabolism in shade stress responses

Based on DEG analysis, some genes encoding key enzymes involved in secondary metabolism were found to be significantly affected by light shading (Kimura et al., 2003), with significant enrichment of genes associated with flavonoid biosynthesis. Catechin content decreases in tea plants under shade stress (Ku et al., 2010; Wang et al., 2012). Previous transcriptome analyses have revealed that genes involved in flavonoid biosynthesis are significantly down-regulated in *Zhonghuang 2* compared with a green tea cultivar. In our study, the expression of genes involved in flavonoid biosynthesis was also repressed in *Baijiguan* following shade treatments (Figure 8B). This repression may be responsible for suppressing photosynthetic pathways, particularly “photosynthesis” and “carbon metabolism” pathways. Other secondary metabolic processes, such as phenylalanine metabolism, phenylpropanoid biosynthesis, and terpenoid backbone biosynthesis, were also significantly enriched (Figure 7). These data suggest that the levels of secondary metabolites in *Baijiguan* were altered; therefore, further studies of the metabolic profile of this cultivar are important.

## Conclusions

Comparative transcriptome analysis of a yellow leaf tea plant variety was performed under shade conditions, and various genes and pathways potentially responsible for yellow leaf formation were identified. GO and pathway enrichment analyses revealed that these DEGs are involved in ROS scavenging system, chloroplast development and photosynthetic pigment synthesis pathways, and that gene expression alterations in these pathways might be responsible for the decreased Chl content and yellow phenotype of *Baijiguan*. Furthermore, the identification of a large number of DEGs offers a global view of light-induced albinism in tea plants, which will aid in the understanding of the molecular mechanisms of yellow leaf formation and will facilitate molecular breeding of tea plants.

## Author contributions

Conceived and designed the experiments: WS, QW, and ZC. Performed the experiments: QW and ZC. Analyzed the data: QW, WS, MC, ZC, and TD. Contributed reagents/materials/analysis tools: QW, ZC, and TD. Contributed to the writing of the manuscript: QW, WS, and MC. Revised and approved the final version of the paper: QW, WS, ZC, MC, and TD.

### Conflict of interest statement

The authors declare that the research was conducted in the absence of any commercial or financial relationships that could be construed as a potential conflict of interest.
