# Supplementary material for: De novo Sequencing of the Leaf Transcriptome Reveals Complex Light-Responsive Regulatory Networks in Camellia sinensis cv. Baijiguan
Source: Front Plant Sci. 2016 Mar 21;7:332. doi: 10.3389/fpls.2016.00332 (PMC4801010; doi:10.3389/fpls.2016.00332)
Supplement: Supplementary file 1 [file DataSheet1.docx]

***Supplementary Material***

***De novo* sequencing of the leaf transcriptome reveals complex light-**

**responsive regulatory networks in *Camellia sinensis* cv. *Baijiguan***

Quanjin Wu *^1^*, Zhidan Chen *^2^*, Weijiang Sun *^1, 2^**, Tingting Deng *^1^*, Mingjie Chen *^3^*

*^1^* Department of Tea Science, College of Horticulture, Fujian Agriculture and Forestry University, Fuzhou, China

*^2^* Department of Tea Science, Anxi College of Tea Science, Fujian Agriculture and Forestry University, Fuzhou, China

*^3^* Haixia Institute of Science and Technology, Fujian Agriculture and Forestry University, Fuzhou, China

***Correspondence:** Weijiang Sun, *^1^* Department of Tea Science, College of Horticulture, *^2^* Department of Tea Science, Anxi College of Tea Science, Fujian Agriculture and Forestry University, No. 15 Shangxiadian Road, Cangshan, Fuzhou, 350002, China. swj8103@126.com

## Supplementary Figures

**
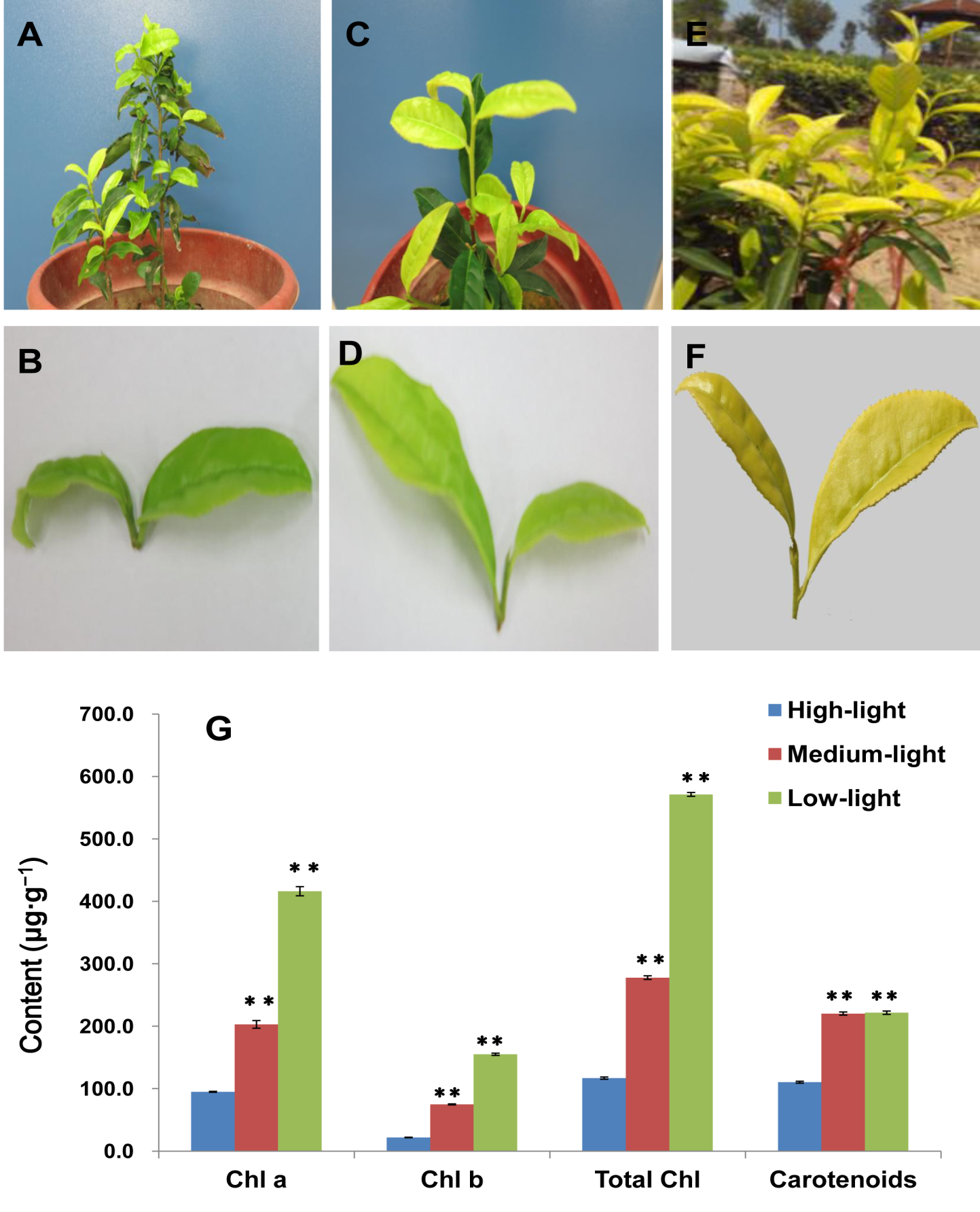
**

**Figure S1 | Phenotype and pigment content in *Baijiguan* under different light intensities. (A,B)** Low-light intensity (240 μmol m^−2^ s^−1^); **(C,D)** medium-light intensity (600 μmol m^−2^ s^−1^); **(E,F)** high-light intensity (1400–1600 μmol m^−2^ s^−1^). **(G)** Pigment content in *Baijiguan*. * represents a significant difference (P < 0.05) between the control and shade treatment. ** represents a highly significant difference (P < 0.01) between the control and shade treatment.


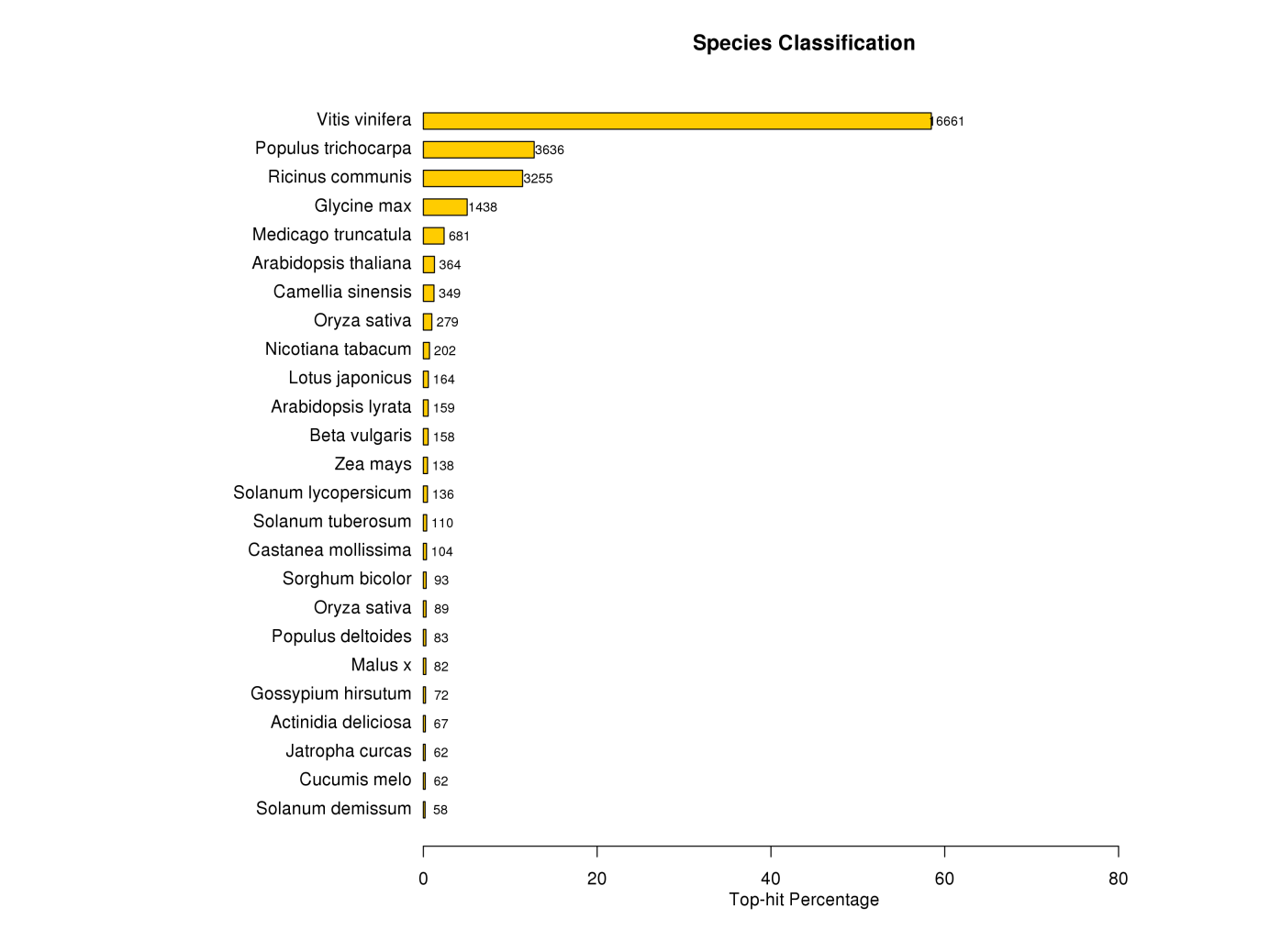


**Figure S2 | Blastx search of the *C. sinensis* transcriptome against the Nr database identified the top 25 species matches with different numbers and percentages of unigenes.**

**
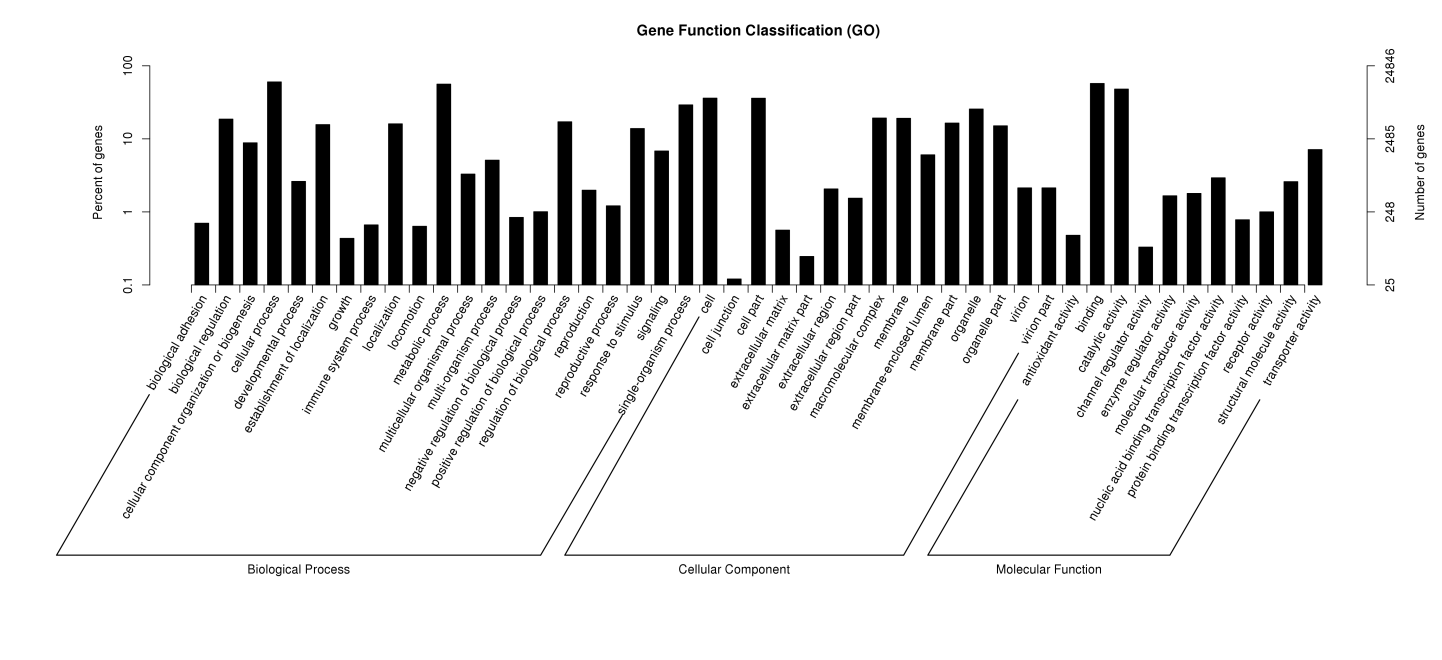
**

**Figure S3 | GO analysis of 24,846 unigenes identified in this study.** All 24,846 unigenes were divided into three functional categories and 47 GO terms. The right Y-axis indicates the number of genes in each category. The left Y-axis shows the percentage of a specific category of genes in each main category.

**
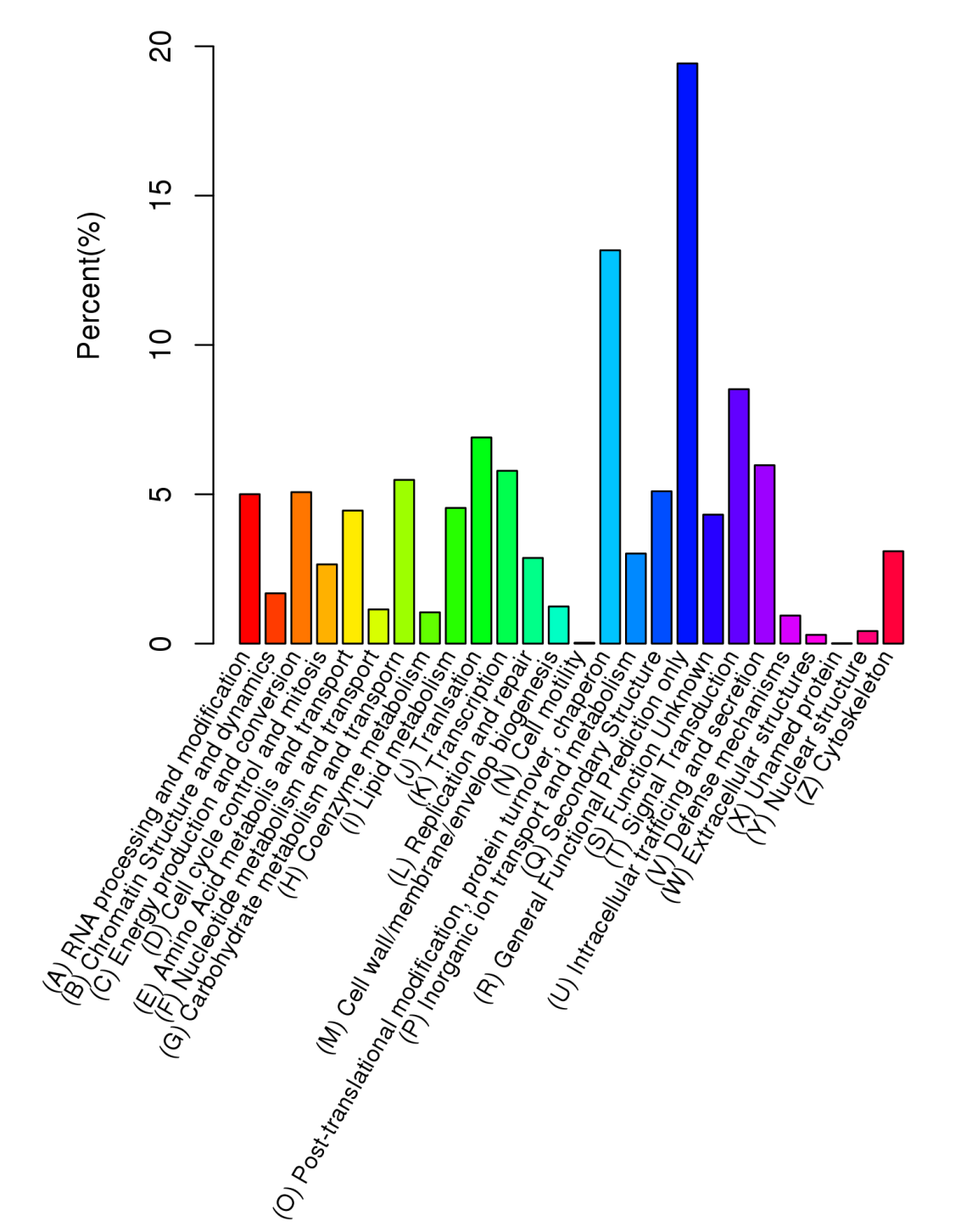
**

**Figure S4 | Classification of the putative proteins by KOG analysis.** All 10,213 unigenes were annotated into 26 classifications. The Y-axis represents the percentage of unigenes that were assigned to a specific functional category.

**
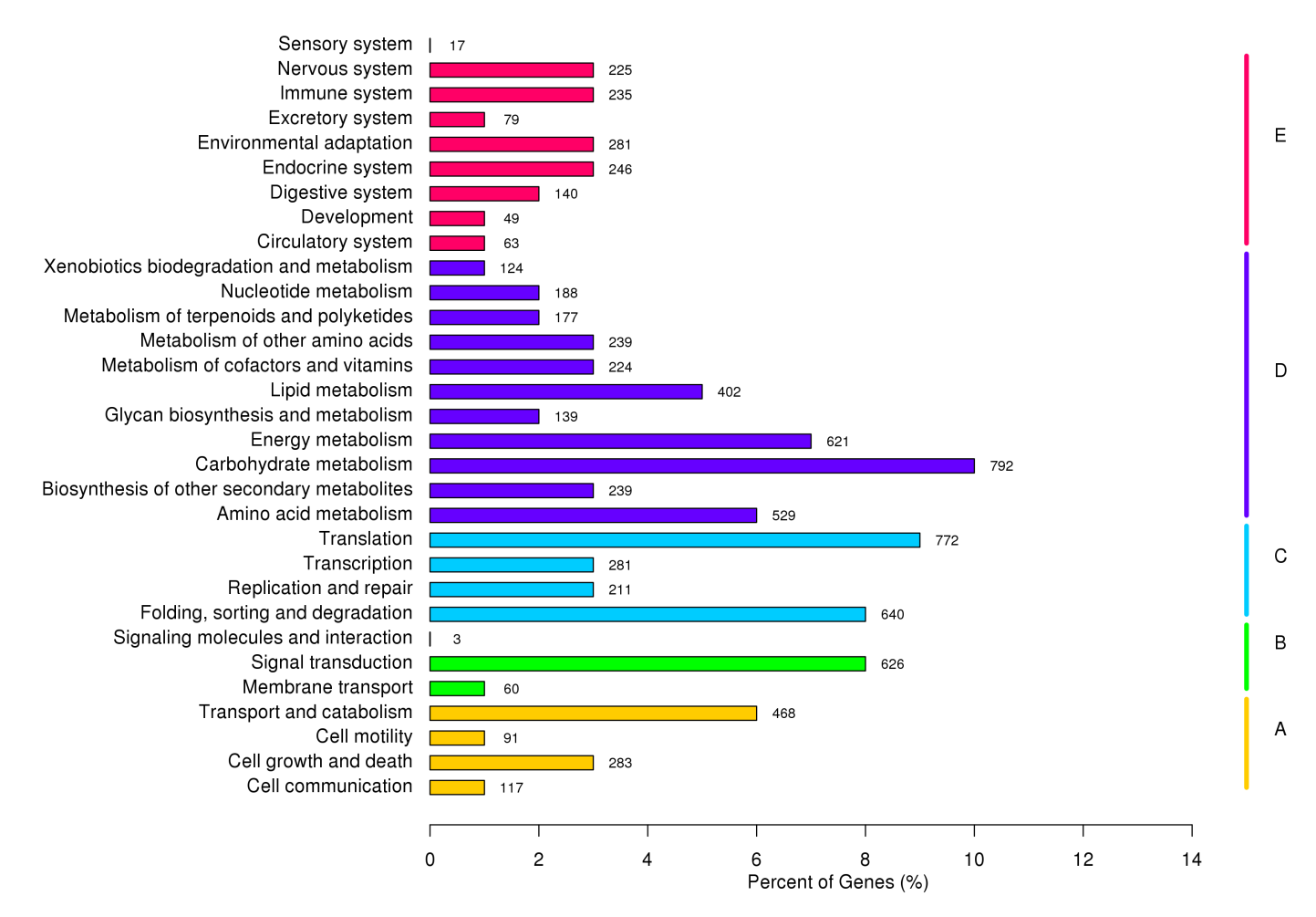
**

**Figure S5 | Histogram of KEGG classifications of the annotated transcripts.** The Y-axis on the left represents the KEGG pathways, and the Y-axis on the right indicates the sub-branches. **(A)** Cellular processes; **(B)** environmental information processing; **(C)** genetic information processing; **(D)** metabolism; and **(E)** organismal systems. The X-axis indicates the percentage of unigenes in a specific pathway.


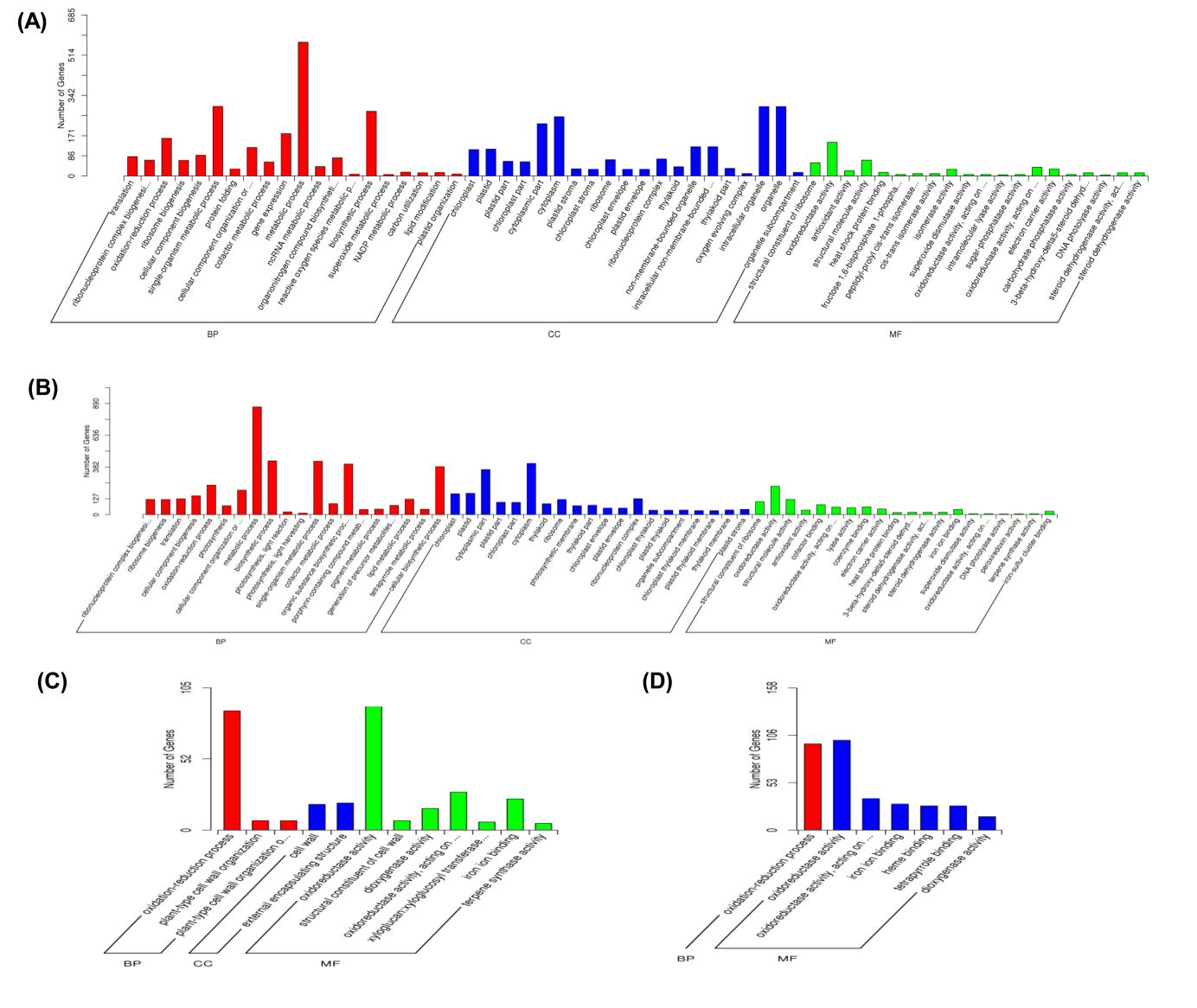


**Figure S6 | GO classifications revealed down- and up- regulated gene categories.** **(A)** Down-regulated gene categories for T3d_Z compared to T3d_W; **(B)** down-regulated gene categories for T6d_Z compared to T6d_W; **(C)** up-regulated gene categories for T3d_Z compared to T3d_W; **(D)** up-regulated gene categories for T6d_Z compared to T6d_W. The Y-axis on the right represents the number of DEGs in a category. The Y-axis on the left indicates the percentage of a specific category of DEGs in each main category.

**Figure S7 | Top 20 enriched and up-regulated KEGG pathways. (A)** T3d_Z vs. control T3d_W; and **(B)** T6d_Z vs. control T6d_W. The Y-axis on the left represents the KEGG pathways, and the X-axis indicates the rich factor. Low q-values are shown in red, and high q-values are depicted in blue.

## Supplementary Tables

The supplementary tables for this article can be found in data sheet 2.

**Table S1 | Primers used for quantitative RT-PCR.**

**Table S2 | Pigment contents in yellow-leaf and green-leaf cultivars.** Data with different letters (a, b) in the same column represent significance at the level of P < 0.01.

**Table S3 | Statistics of DEGs at different time points.** Clean reads for the samples were mapped to the reference sequences derived from our *C. sinensis* transcriptome data.

**Table S4 | Differentially co-expressed genes of the T6d_Z vs. T6d_W group compared to the T3d_Z vs. T3d_W group.**

**Table S5 | DEGs encoding putative TFs in *C. sinensis* were identified between both the T3d_Z vs. T3d_W group and the T6d_Z vs. T6d_W group.**

**Table S6 | DEGs for the T6d_Z vs. the T3d_Z group.**

**Table S7 Unigenes involved in the top 20 enriched KEGG pathways.**
